# Valproic acid remodels phosphatidylglycerol homeostasis and epithelial-macrophage crosstalk in inflammatory signaling

**DOI:** 10.1016/j.isci.2026.117369

**Published:** 2026-09-04

**Authors:** Ivana Ďurišová, Mariana Máčajová, Ingrid Zriniová, Ivana Fábryová, Mailinda Meta, Lenka Bábelová, Boris Bilčík, Aleš Kvasnička, Miroslav Baláž, Soňa Bernátová, Silvia Petrezsélyová, Dominik Filipp, Yuan-Hao Hsu, David Friedecký, Ivan Čavarga, Mária Balážová

**Affiliations:** 1 Department of Membrane Biochemistry, Centre of Biosciences, Slovak Academy of Sciences, Institute of Animal Biochemistry and Genetics, Bratislava, Slovakia; 2 Department of Physiology and Ethology, Centre of Biosciences, Slovak Academy of Sciences, Institute of Animal Biochemistry and Genetics, Bratislava, Slovakia; 3 Department of Clinical Biochemistry, University Hospital Olomouc and Faculty of Medicine and Dentistry, Palacky University Olomouc, Olomouc, Czechia; 4 Department of Medical Biochemistry, Oslo University Hospital, Oslo, Norway; 5 Institute of Experimental Endocrinology, Biomedical Research Center, Slovak Academy of Sciences, Bratislava, Slovakia; 6 Department of Animal Physiology and Ethology, Faculty of Natural Sciences, Comenius University, Bratislava, Slovakia; 7 Institute of Medical Biology, Genetics and Clinical Genetics, Faculty of Medicine, Comenius University, Bratislava, Slovakia; 8 Laboratory of Immunobiology, Institute of Molecular Genetics of the Czech Academy of Sciences, Prague, Czech Republic; 9 Department of Chemistry, Tunghai University, Taichung, Taiwan

**Keywords:** lipid metabolism, phosphatidylglycerol, valproic acid, inflammatory signaling

## Abstract

Pulmonary surfactant lipids support lung homeostasis and innate immunity, and their dysregulation contributes to acute respiratory distress syndrome. Whether mitochondrial phosphatidylglycerol (PG) biosynthesis influences epithelial lipid secretion and inflammatory responses remains unclear. Using A549 cells, we disrupted *PGS1*, which encodes phosphatidylglycerophosphate synthase, and found that partial loss of *PGS1* altered cellular PG levels and changed the secreted phospholipid profile. Valproic acid (VPA), a clinically used antiepileptic drug, similarly remodeled secreted lipids and increased the relative abundance of the anti-inflammatory PG species palmitoyl-oleoyl-phosphatidylglycerol (POPG), an effect not seen with partial *PGS1* loss. Functionally, conditioned media from VPA-treated epithelial cells reduced lipopolysaccharide-induced inflammatory responses in PMA-differentiated U937 macrophage-like cells, with lipid extracts contributing to this effect. In a vascularized quail chorioallantoic membrane model, both VPA and POPG decreased inflammation and maintained tissue structure. Overall, these findings connect mitochondrial PG metabolism to epithelial lipid-mediated control of inflammation.

## Highlights

• Mitochondrial PG homeostasis regulates epithelial lipid secretion
• VPA remodels PG metabolism and increases anti-inflammatory POPG
• Epithelial lipids mediate anti-inflammatory epithelial-macrophage crosstalk
• VPA and POPG suppress inflammation in a vascularized CAM model

## Graphical abstract

**Figure:**
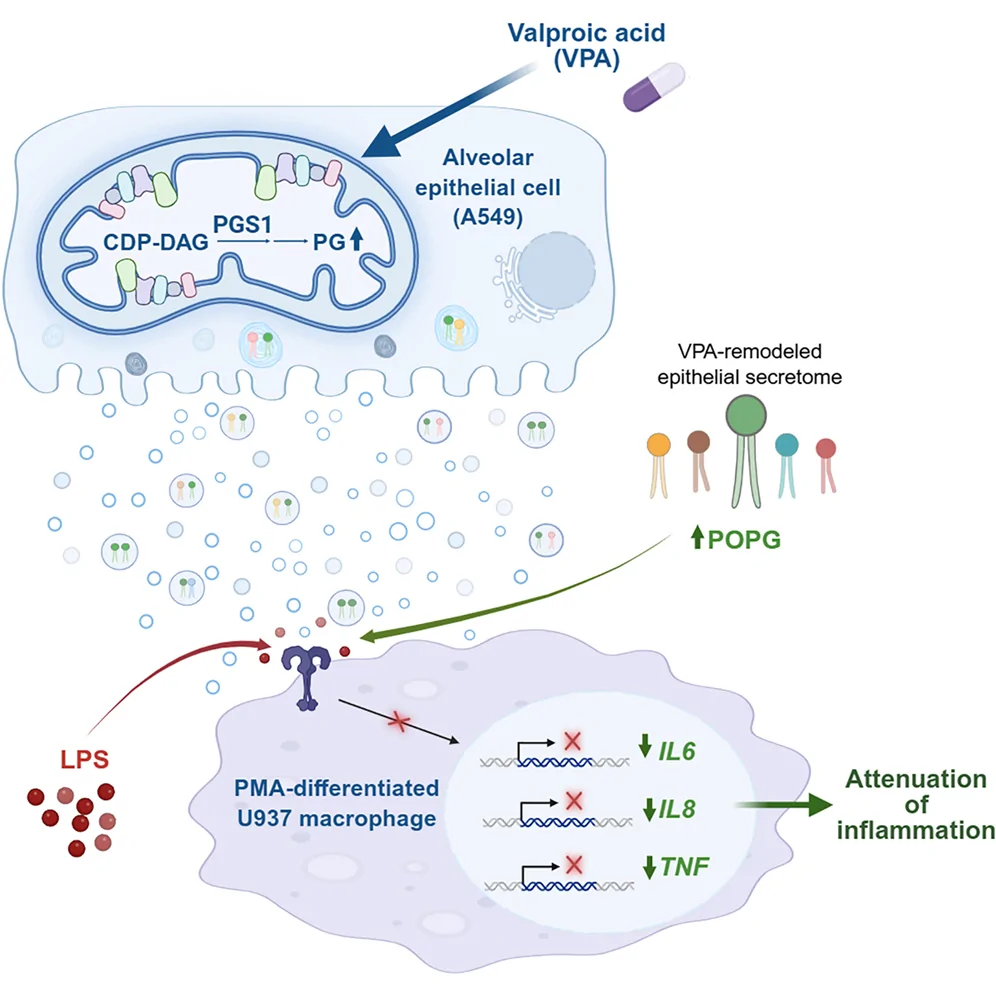

## Teaser

Biological sciences; Human metabolism; Immunology; Lipidomics

## Introduction

Pulmonary surfactant, produced by alveolar type II (ATII) cells, plays a key role in maintaining alveolar stability and regulating lung immunity.^1^^,^^2^^,^^3^^,^^4^^,^^5^^,^^6^ Among surfactant lipids, phosphatidylglycerol (PG) has emerged as an anti-inflammatory component. Its major molecular species, palmitoyl-oleoyl-PG (POPG), dampens Toll-like receptor 4 (TLR4) signaling by limiting the transfer and recognition of lipopolysaccharide (LPS) within the CD14-MD2-TLR4 pathway. As a consequence, POPG reduces inflammation and tissue injury in experimental models.^7^^,^^8^^,^^9^^,^^10^^,^^11^^,^^12^^,^^13^ Reduced alveolar PG is observed in acute respiratory distress syndrome (ARDS) and correlates with impaired surfactant function and dysregulated innate immunity,^14^^,^^15^^,^^16^^,^^17^ highlighting the importance of maintaining adequate PG for lung homeostasis. Strategies aimed at restoring alveolar PG levels, including exogenous POPG delivery or surfactant supplementation, have shown promise in experimental models of inflammation.^8^^,^^9^^,^^18^^,^^19^^,^^20^^,^^21^

Beyond its extracellular role in the alveolar space, intracellular PG also contributes to mitochondrial membrane organization. Together with cardiolipin (CL), PG supports respiratory chain function and mitochondrial dynamics, which in turn shape innate immune programs through metabolic and redox signaling.^22^^,^^23^^,^^24^^,^^25^ However, it remains unclear how mitochondrial PG homeostasis is linked to the composition of surfactant-associated lipids released by epithelial cells and to downstream epithelial-immune signaling.

PG and CL biosynthesis predominantly occur at the inner mitochondrial membrane, where the rate-limiting step is catalyzed by phosphatidylglycerophosphate synthase 1 (PGS1). PGS1 activity is sensitive to *myo*-inositol availability, which shifts phospholipid synthesis toward phosphatidylinositol (PI) at the expense of PG.^26^^,^^27^^,^^28^^,^^29^^,^^30^ Valproic acid (VPA), a widely used antiepileptic and mood stabilizer, modulates mitochondrial metabolism and inositol homeostasis.^31^^,^^32^^,^^33^^,^^34^ Consistent with this pleiotropy, VPA has been reported to remodel mitochondrial phospholipid pathways, including PG/CL-related biosynthesis, in a context-dependent manner.^35^^,^^36^^,^^37^ However, it remains unclear whether VPA-induced remodeling of mitochondrial lipid metabolism translates into altered surfactant-associated lipid secretion and subsequent modulation of inflammatory signaling in lung epithelial systems.

Here, we examine the role of mitochondrial PG biosynthesis in controlling surfactant-associated lipid secretion and inflammatory responses in lung epithelial cells by combining genetic modulation of *PGS1* with pharmacological treatment using VPA. We further test how VPA-driven epithelial lipid remodeling influences macrophage activation using conditioned media and evaluate its impact on inflammatory responses in the densely vascularized Japanese quail chorioallantoic membrane (CAM) model.^38^ By integrating mitochondrial lipid biochemistry, epithelial secretory lipidomics, and immune readouts, we ask whether alterations in mitochondrial PG biosynthesis are sufficient to reprogram epithelial-macrophage crosstalk and tissue-level inflammatory responses. Together, our data support a model in which pharmacological and genetic remodeling of mitochondrial phospholipid homeostasis is linked to changes in epithelial secretory cues that modulate inflammatory signaling.

## Results

### Mitochondrial PG biosynthesis controls phospholipid secretion and metabolic activity in A549 cells

A549 cells serve as a well-established epithelial model for studying surfactant lipid metabolism and alveolar lipid homeostasis. Using this system, we asked whether changes in mitochondrial PG biosynthesis are reflected in the quantity and molecular composition of secreted phospholipids. To assess whether mitochondrial PG biosynthesis affects epithelial phospholipid secretion and cellular metabolism, we genetically and pharmacologically altered the rate-limiting enzyme encoded by *PGS1*. This was achieved by (i) generating a heterozygous *PGS1* mutant and (ii) treating cells with VPA, a compound known to interfere with mitochondrial lipid metabolism in both mammalian cells and yeast.^35^^,^^36^^,^^37^

To prepare a *PGS1* mutant A549 cell line, we employed CRISPR-Cas9 tools targeting exon 2 of the gene (Figure S1A). By this approach, we were able to prepare a heterozygous *PGS1* cell line (termed as *PGS1*^+/−^). We obtained only mono-allelic disruption, consistent with reduced viability/fitness upon stronger impairment of PG/CL pathways reported in other systems.^39^^,^^40^ The selected *PGS1*^+/−^ clone harbored a 13-nucleotide deletion in one allele (Figures S1A and S1B), which is predicted to truncate *PGS1* upstream of its conserved catalytic motifs (HKD), consistent with loss of enzymatic activity.^41^ Due to the lack of a suitable antibody, the gene disruption was validated by Sanger sequencing and by RT-qPCR using primers that span the deleted exon 2 region, showing the loss of mRNA (Figures S1B and S1C).

**Figure 1:**
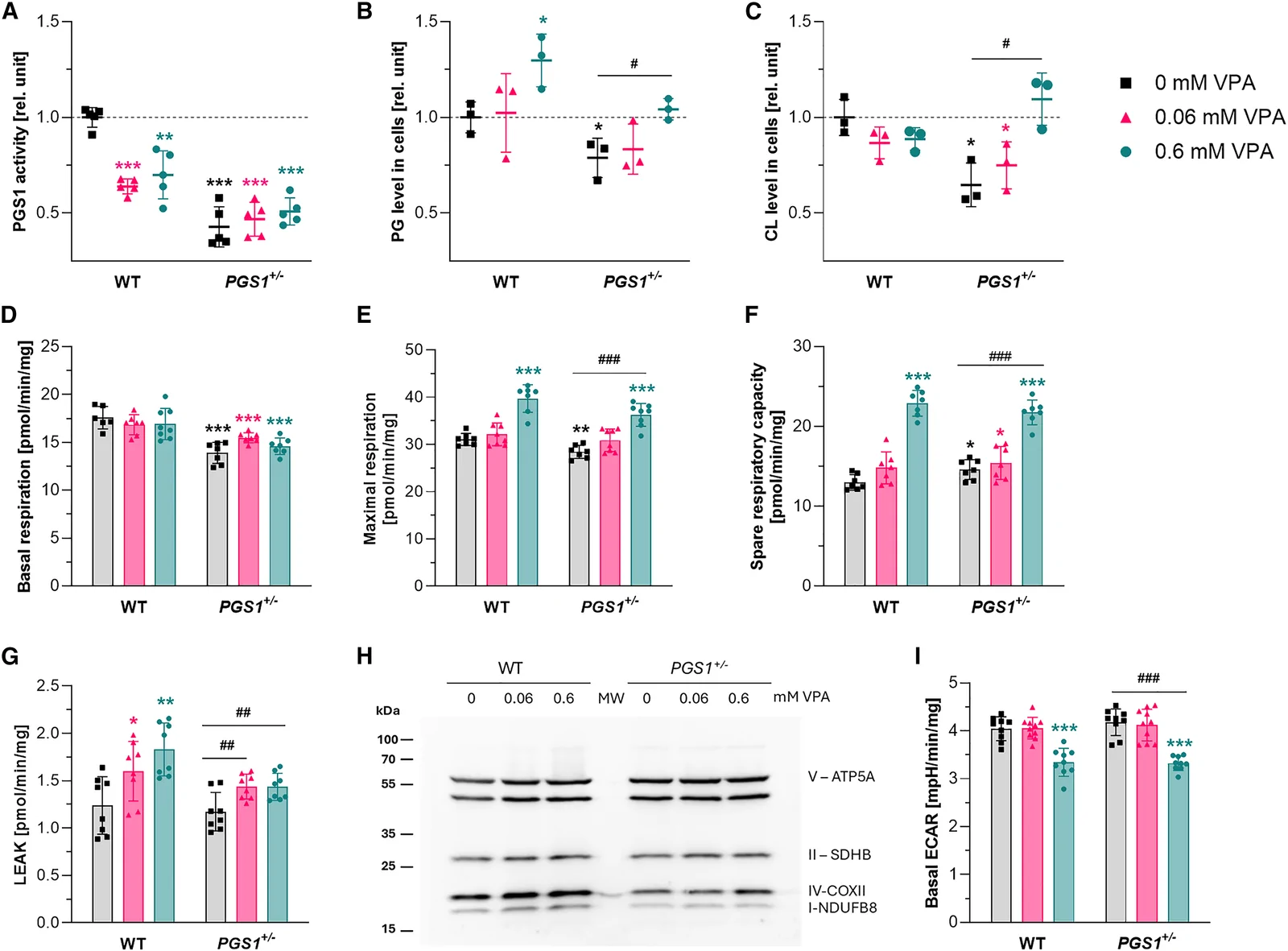
VPA reprograms mitochondrial lipid composition and metabolic output in A549 cells WT and *PGS1*^+/−^ A549 cells were cultured for 5 days and then treated for an additional 2 days with 0, 0.06, or 0.6 mM VPA in serum-free DMEM. (A) PGS1 activity measured in cell homogenates. (B) Cellular PG and (C) CL levels determined by phosphate assay. (D) Basal oxygen consumption rate in intact cells as an indicator of mitochondrial respiration. (E) Maximal respiration after addition of the uncoupler FCCP. (F) Spare respiratory capacity calculated as the difference between maximal and basal respiration. (G) Proton leak (LEAK) after addition of oligomycin. (H) Representative immunoblot showing respiratory chain subunits NDUFB8 (complex I), SDHB (complex II), UQCRC2 (complex III), COXII (complex IV), and ATP5A (ATP synthase). (I) Basal ECAR. Data represent mean ± SD from at least three independent experiments (individual data points shown). (A–C) are normalized to untreated WT cells (0 mM VPA). Statistical significance was assessed using one-way ANOVA followed by the Holm-Šidák multiple-comparisons test. Asterisks indicate significant differences relative to untreated WT; hashtags denote significant differences among VPA concentrations within the same cell line. ∗*p* < 0.05, ∗∗*p* < 0.01, ∗∗∗*p* < 0.001 or ^#^*p* < 0.05, ^##^*p* < 0.01, ^###^*p* < 0.001. Key legend is for (A–G) and (H).

Importantly, heterozygous loss of *PGS1* was sufficient to reduce its enzymatic activity by 57% (Figure 1A, 0 mM VPA), accompanied by a 22% and 35% decrease in membrane PG and CL, respectively (Figures 1B and 1C). Analysis of additional intracellular phospholipid classes, normalized to cellular protein, revealed that *PGS1*^*+/−*^ cells also exhibited reduced total phospholipid and PC content, whereas PE and PI + PS remained comparable to WT cells (Figure S2A). Despite these changes, the relative distribution of phospholipid classes was largely preserved, with CL being the only phospholipid class that was significantly reduced (Figure S2B). Reduced cellular PG and CL levels correlated with impaired mitochondrial function, as evidenced by a 12% decrease in basal and an 8% decrease in maximal oxygen consumption rates (Figures 1D–1F) and reduced abundance of respiratory chain subunits NDUFB8 (complex I) and COXII (complex IV) (Figure 1H; Figure S3). These data indicate that a partial loss of *PGS1* is sufficient to compromise mitochondrial function in A549 cells.

Subtherapeutic and therapeutic concentrations of VPA (0.06 and 0.6 mM) did not affect cell proliferation in either WT or *PGS1*^+/−^ cells within 48 h. In wild-type (WT) A549 cells, VPA decreased PGS1 activity by up to 30% (Figure 1A). In parallel, membrane PG levels increased (Figures 1B and 1C). This pattern is consistent with our previous observations following VPA treatment.^36^^,^^37^ In *PGS1*^+/−^ cells, VPA restored PG and CL to near WT levels, while PGS1 activity remained comparable to untreated *PGS1*^+/−^ cells (Figures 1A–1C). Concomitantly, VPA restored intracellular total phospholipid and PC levels in *PGS1*^+/−^ cells to values comparable to those of untreated WT cells (Figure S2A).

While VPA did not significantly alter basal respiration, treatment with 0.6 mM VPA strongly increased maximal respiration, spare respiratory capacity, and LEAK respiration in both cell lines (Figures 1D–1G). In WT cells, these effects were accompanied by increased abundance of complexes I, II, IV and ATP synthase subunits, whereas in *PGS1*^+/−^ cells only complex IV subunit was modestly increased (Figure 1H; Figure S3). Concomitantly, basal extracellular acidification rate (ECAR) was reduced by 17% in WT and by 20% in *PGS1*^+/−^ cells, indicating a metabolic shift away from glycolysis toward enhanced mitochondrial reliance (Figure 1I). Together, these data support that VPA enhances mitochondrial respiratory capacity while reshaping the lipid composition of mitochondrial membranes.

**Figure 2:**
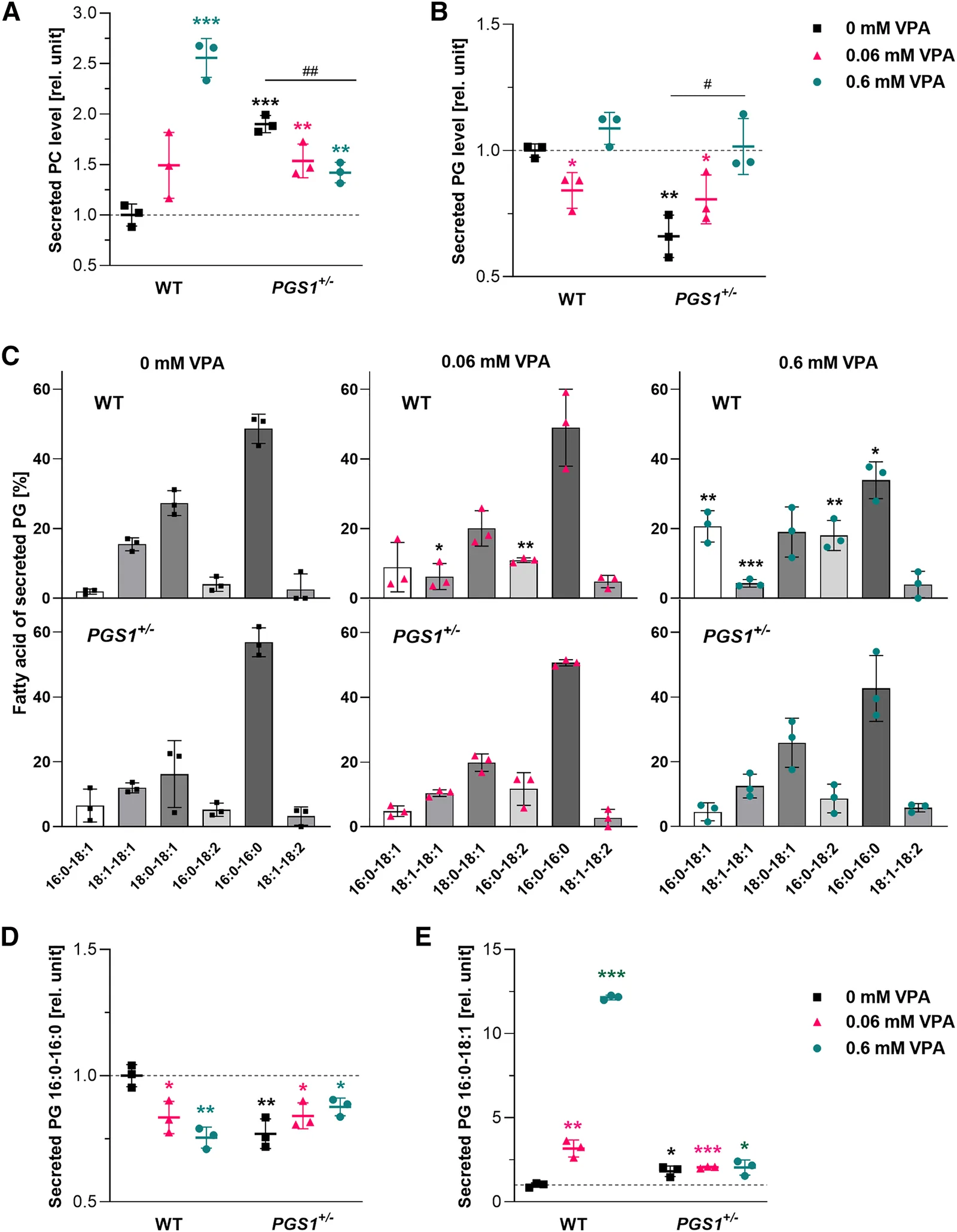
Biosynthesis of PG and VPA treatment affect the quality and quantity of secreted phospholipids in A549 cells Culture supernatants of WT and *PGS1*^+/−^ A549 cells were collected, cleared by centrifugation, and analyzed by mass spectrometry. Secretion levels of (A) PC and (B) PG are shown. (C) Relative fatty acyl chain composition of secreted PG. Levels of (D) dipalmitoyl PG (16:0-16:0) and (E) palmitoyl-oleyl PG (16:0-18:1) in the medium are shown. Data represent mean ± SD from three independent experiments (individual points shown). (A, B, D, and E) are normalized to untreated WT cells (0 mM VPA). Statistical significance was assessed using one-way ANOVA followed by the Holm-Šidák multiple-comparisons test. Asterisks indicate significant differences relative to untreated WT cells; hashtags denote significant differences among VPA concentrations within the same cell line. ∗*p* < 0.05, ∗∗*p* < 0.01, ∗∗∗*p* < 0.001, ^#^*p* < 0.05, ^##^*p* < 0.01.

Next, we examined how altered PGS1 activity and VPA treatment affected the secretion of PC and PG, the two major surfactant phospholipids. *PGS1*^+/−^ cells exhibited a ∼2-fold increase in PC secretion compared to WT A549 cells. In WT cells, VPA increased PC secretion in a dose-dependent manner (1.5- and 2.5-fold at 0.06 and 0.6 mM, respectively). In contrast, 0.6 mM VPA reduced PC secretion by ∼25% in *PGS1*^+/−^ cells (Figure 2A). Reduced PGS1 activity also caused a 34% decrease in PG secretion relative to WT A549 cells (Figure 2B). While VPA had only a minimal effect on PG secretion in WT, 0.6 mM VPA restored PG secretion in *PGS1*^+/−^ cells to WT levels.

Although POPG is the most abundant PG species in native pulmonary surfactant,^42^ both WT and *PGS1*^+/−^ cells mainly secreted dipalmitoyl-PG (DPPG; C16:0–C16:0), accounting for 49% and 57% of total PG, respectively, whereas POPG represented only ∼2% and 6.5%, respectively (Figure 2C). While the PG fatty acid profile remained stable across time and cultivation conditions, VPA markedly altered PG composition in WT cells: DPPG decreased by 25%, whereas POPG increased 12-fold. No significant compositional changes were observed in *PGS1*^+/−^ cells, indicating that intact PGS1 activity is required for VPA-induced PG remodeling (Figures 2C–2E).

Analysis of additional lipid classes revealed that VPA broadly altered lipid secretion patterns in WT A549 cells, affecting both the abundance and relative ratios of several lipid species (Figures S4A and S4B). These alterations were largely absent in *PGS1*^+/−^ cells, except for plasmalogens and diacylglycerol (DAG), supporting a central role for the PGS1-dependent pathway in coordinating VPA-driven lipid secretion. In summary, these data suggest that mitochondrial PG biosynthesis, controlled by the *PGS1*-encoded enzyme and modulated by VPA, governs both the quantity and molecular composition of secreted phospholipids and is tightly linked to cellular metabolic reprogramming in A549 cells.

### Regulation of inflammation in A549 cells

**Figure 3:**
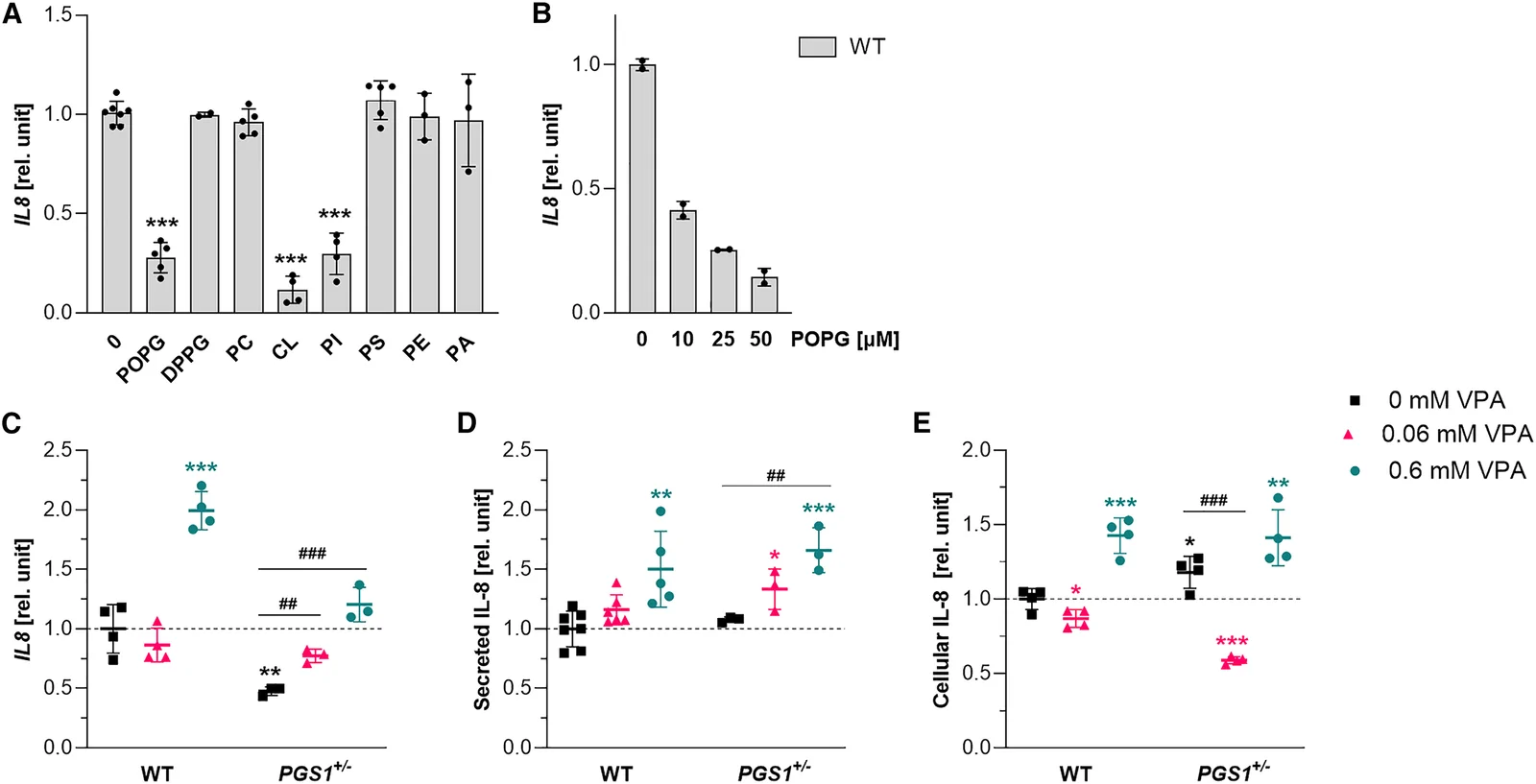
*IL8* expression is modulated by phospholipids and VPA treatment in epithelial cells Inflammation in WT and *PGS1*^+/−^ A549 cells was induced by the addition of 1 μg/mL LPS to the culture medium. (A) *IL8* expression in cells stimulated with LPS in the presence or absence of exogenous phospholipids. (B) *IL8* expression in cells treated with increasing concentrations of POPG. (C) *IL8* expression, (D) secreted IL-8, and (E) cellular IL-8 in cells pretreated with VPA and subsequently stimulated with LPS. Data represent mean ± SD from at least two independent experiments (individual points shown) and are normalized to untreated WT cells (0 mM VPA). Statistical significance was assessed using one-way ANOVA followed by the Holm-Šidák multiple-comparisons test. Asterisks indicate significant differences relative to untreated WT cells; hashtags denote significant differences among VPA concentrations within the same cell line. ∗*p* < 0.05, ∗∗*p* < 0.01, ∗∗∗*p* < 0.001, ^##^*p* < 0.01, ^###^*p* < 0.001. CL, cardiolipin; DPPG, dipalmitoyl phosphatidylglycerol; PA, phosphatidic acid; PC, phosphatidylcholine; PE, phosphatidylethanolamine; PI, phosphatidylinositol; PS, phosphatidylserine; VPA, valproic acid.

To quantify epithelial inflammatory responses, we used *IL8* expression as a commonly used readout of LPS stimulation in A549 cells.^43^ In this model, induction of other cytokines, such as *IL6*, can require additional priming or co-stimulation, as LPS alone has been reported to be insufficient.^44^ Inflammation in WT and *PGS1*^+/−^ cells was induced by the addition of 1 μg/mL LPS to the culture medium. To test whether exogenous phospholipids modulate epithelial inflammatory responses, cells were stimulated with LPS in the presence or absence of selected phospholipids, and *IL8* expression was quantified (Figure 3A). Co-treatment with POPG, PI, or CL significantly attenuated LPS-induced *IL8* expression compared to controls, whereas DPPG, PC, phosphatidic acid (PA), phosphatidylethanolamine (PE), or phosphatidylserine (PS) did not (Figure 3A). The inhibitory effect of POPG was dose-dependent (Figure 3B), consistent with competitive antagonism of LPS signaling.^13^

Next, we examined how reduced mitochondrial PG biosynthesis affects epithelial inflammatory signaling. Basal *IL8* mRNA levels were higher in unstimulated *PGS1*^+/−^ cells compared to WT A549 cells. Despite this, LPS-induced *IL8* upregulation was reduced by ∼50% in *PGS1*^+/−^ cells relative to WT cells (Figure 3C; Figures S5A and S5B). In contrast, IL-8 protein secretion under basal and LPS-stimulated conditions was not significantly different between both cell lines (Figure 3D; Figure S5C). To complement the analysis of secreted IL-8, cellular IL-8 protein levels were quantified. A modest increase in cellular IL-8 protein was observed in LPS-stimulated *PGS1*^+/−^ cells compared to WT cells (Figure 3E). Thus, heterozygous loss of *PGS1* altered the coordination between *IL8* transcription and IL-8 protein responses.

We then tested the impact of VPA pretreatment on epithelial inflammatory responses. VPA treatment alone increased basal *IL8* expression (Figure S5B). Upon LPS stimulation, VPA-pretreated WT and *PGS1*^+/−^ cells showed approximately 2-fold and 2.5-fold higher *IL8* mRNA levels, respectively, compared to untreated cells. While 0.06 mM VPA increased IL-8 secretion despite reduced intracellular IL-8 levels, pretreatment with 0.6 mM VPA increased both intracellular and secreted IL-8 levels (Figures 3D and 3E).

Together, these data support that specific lipid species (POPG, PI, and CL) effectively suppress LPS-induced *IL8* mRNA expression in A549 cells, while VPA-induced metabolic remodeling enhances cellular responsiveness to inflammatory stimuli, resulting in increased IL-8 production.

### Regulation of inflammation in activated monocytes

**Figure 4:**
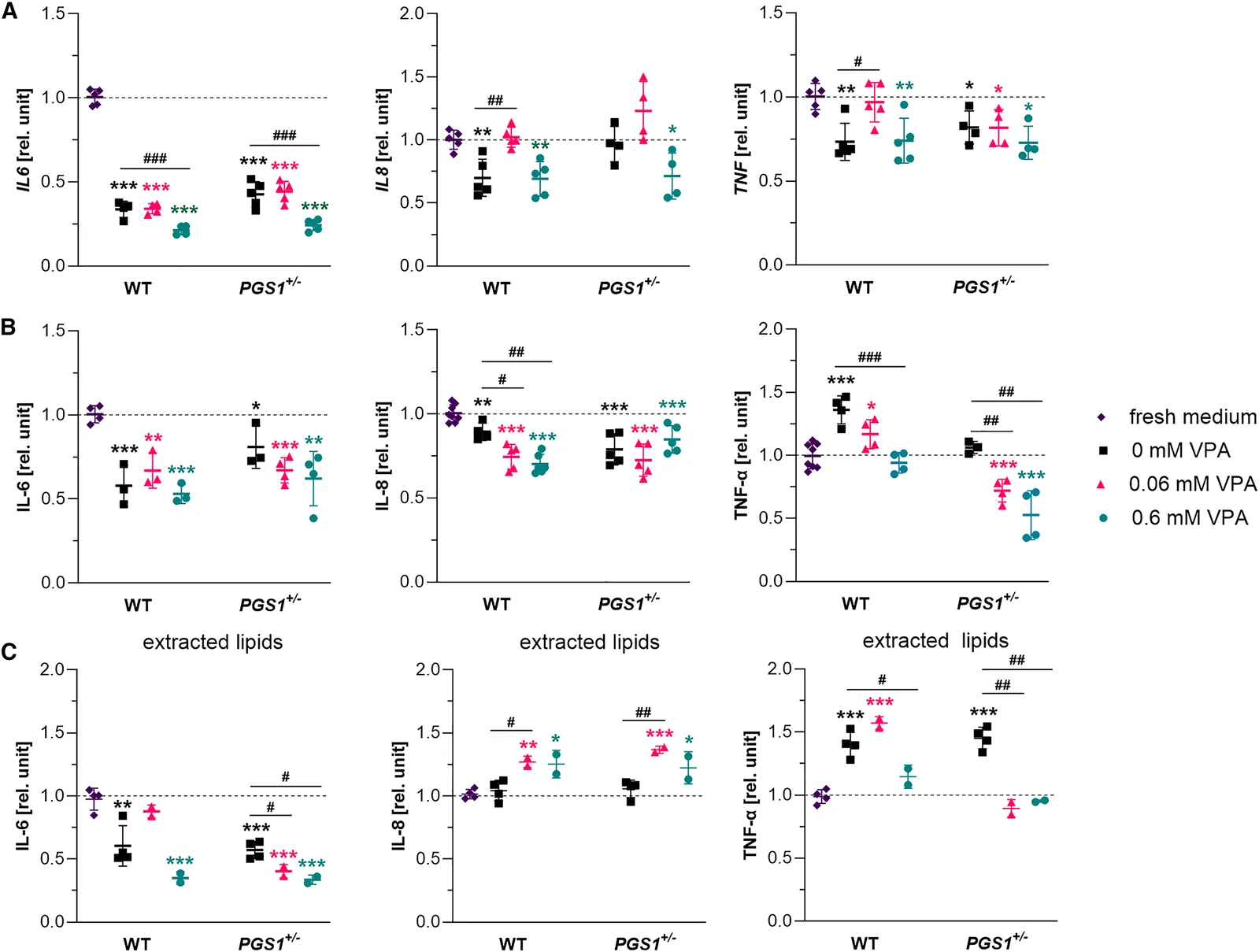
Conditioned media from A549 epithelial cells modulate the inflammatory response of U937 macrophage-like cells Conditioned media from WT and *PGS1*^+/−^ A549 cells pretreated for 48 h with 0, 0.06, or 0.6 mM VPA were cleared by centrifugation and applied to PMA-differentiated U937 cells. Inflammation was subsequently induced by 0.1 μg/mL LPS for 6 h. (A) Relative mRNA expression of *IL6*, *IL8*, and *TNF* in U937 cells. (B) Protein levels of IL-6, IL-8, and TNF-α in U937 supernatants after incubation with conditioned media. (C) Protein levels of IL-6, IL-8, and TNF-α in U937 cells stimulated with LPS in the presence of lipids extracted from conditioned media. Data represent mean ± SD from at least two independent experiments (individual points shown) and are normalized to U937 cells cultured in fresh medium. Statistical significance was assessed using one-way ANOVA followed by the Holm-Šidák multiple-comparisons test. Asterisks indicate significant differences relative to U937 cells in fresh medium; hashtags denote significant differences among VPA concentrations within the same conditioned medium. ∗*p* < 0.05, ∗∗*p* < 0.01, ∗∗∗*p* < 0.001, ^#^*p* < 0.05, ^##^*p* < 0.01, ^###^*p* < 0.001. VPA, valproic acid.

Given that perturbation of mitochondrial PG biosynthesis (via heterozygous loss of *PGS1* and/or VPA treatment) altered the lipid secretory output of A549 cells (Figure 2) and modulated epithelial inflammatory readouts (Figure 3), we next tested whether epithelial conditioned media affect macrophage-like inflammatory responses. For U937 macrophages, we used PMA to differentiate monocytes into macrophage-like cells.^45^ Conditioned media were collected from WT and *PGS1*^+/−^ A549 cells after 48 h of treatment with VPA (0, 0.06, or 0.6 mM). These media were transferred to PMA-differentiated U937 cells, followed by stimulation with 0.1 μg/mL LPS for 6 h (Figure 4).

Conditioned media from VPA-treated WT and *PGS1*^+/−^ epithelial cells exhibited anti-inflammatory effects on U937 macrophages in a concentration-dependent manner (Figures 4A and 4B). To examine lipid-specific contributions, lipids extracted from conditioned media were applied to U937 macrophages. LC-MS analysis of a representative lipid extract prepared from conditioned medium of untreated (0 mM VPA) WT A549 cells confirmed the presence of the major phospholipid classes, including approximately 2.3 μg PC, 50 ng total PG, and 0.96 ng POPG. These lipid extracts partially recapitulated the anti-inflammatory effects of whole conditioned media. Lipids from VPA-treated A549 cells similarly reduced IL-6 and TNF-α, whereas IL-8 secretion showed a modest increase, consistent with the elevated *IL8* transcription (Figure 4C; Figure S6A). To determine whether these effects could be reproduced by defined lipid species, exogenous phospholipids were applied to LPS-stimulated, PMA-differentiated U937 cells. CL, POPG, and PI reduced pro-inflammatory gene expression in a concentration-dependent manner, with higher lipid concentrations exerting stronger suppressive effects (Figures S6B and S6C). These findings support a primary role for lipids in mediating the anti-inflammatory activity of the epithelial secretome.

To assess whether IL-8 released from A549 cells contributes to the effects of conditioned media, recombinant IL-8 was added to LPS-stimulated U937 macrophages. The resulting cytokine profile differed from that induced by conditioned media, indicating that IL-8 alone does not reproduce the suppressive effects observed with epithelial conditioned media (Figures S7A and S7B). Finally, the contribution of residual VPA appeared minimal. Only a modest similarity with conditioned media was observed for IL-8, whereas IL-6 and TNF-α responses did not correlate with direct VPA exposure (Figures S7D–S7F).

Taken together, these data show that A549-conditioned media suppress LPS-induced inflammatory outputs in PMA-differentiated U937 macrophage-like cells. Secreted epithelial lipids contribute to this activity, as lipid extracts reproduce part of the effect, while additional components in conditioned media are likely required for full suppression.

### Regulation of inflammation in the CAM of Japanese quail

While experiments in A549 epithelial cells and PMA-differentiated U937 monocytes provided valuable insight into lipid-mediated modulation of inflammation, both systems represent simplified *in vitro* models. To assess these effects in a more physiologically relevant context, the highly vascularized CAM of Japanese quail embryos cultured *ex ovo* was used as an *in vivo* model of local inflammation.

**Figure 5:**
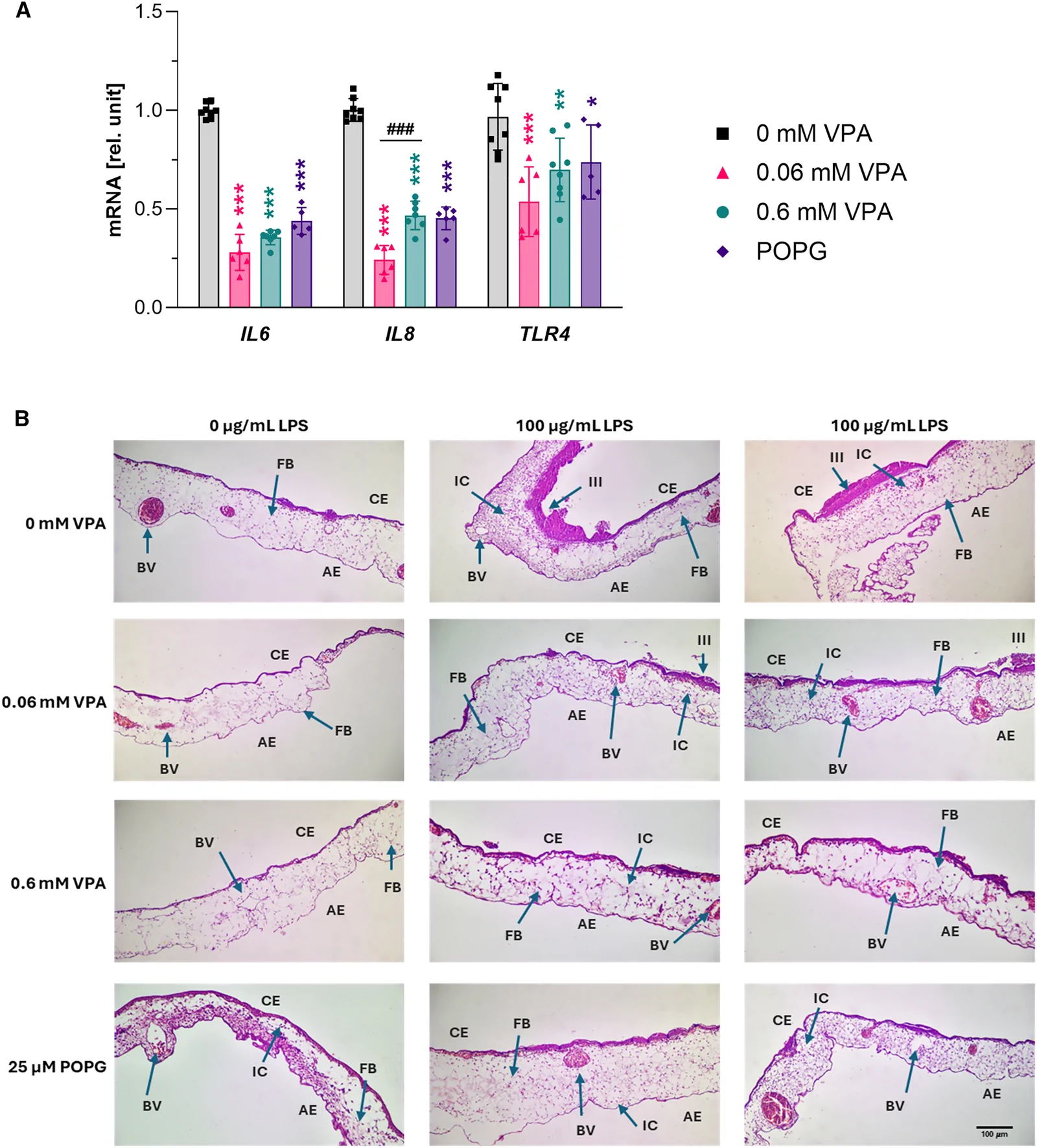
VPA and POPG modulate inflammatory responses in the quail CAM Quail CAMs were pretreated with 0, 0.06, or 0.6 mM VPA for 24 h. Subsequently, inflammation was induced by 100 μg/mL LPS or by 100 μg/mL LPS supplemented with 25 μM POPG for 2 h. (A) Relative mRNA expression of *IL6*, *IL8*, and *TLR4* in CAM tissue within the area defined by the silicone ring. Data are normalized to untreated CAM (0 mM VPA) and shown as mean ± SD from at least five independent experiments (individual points shown). Statistical significance was assessed using one-way ANOVA followed by the Holm-Šidák multiple-comparisons test. Asterisks indicate significant differences relative to untreated CAM; hashtags denote significant differences among VPA concentrations. (B) Representative histological sections stained with hematoxylin and eosin. Scale bars, 100 μm ∗*p* < 0.05, ∗∗*p* < 0.01, ∗∗∗*p* < 0.001, ^###^*p* < 0.001.

We first established LPS conditions that reproducibly trigger inflammation while preserving embryo viability. Embryo survival was not significantly affected at concentrations up to 800 μg/mL, whereas 1600 μg/mL reduced survival to approximately 50% (Figure S8A). Histological alterations were already evident at 100 μg/mL LPS, including increased cellularity, stromal edema, thickening of the chorionic epithelium, and accumulation of immune cells within the mesenchyme (Figure 5; Figure S8A). In contrast, quantitative vascularization parameters (vessel area, vessel length, branching points, and mean vessel thickness) remained largely unchanged under these conditions (Figure S8B).

Based on these pilot experiments, we selected 100 μg/mL LPS for subsequent analyses. We then assessed inflammatory gene expression, focusing on *IL6*, *IL8*, and *TLR4*. LPS induced all three transcripts (Figure S9). Pretreatment with VPA at both tested concentrations significantly attenuated this induction (Figure 5A), with the strongest effect observed at 0.06 mM, reducing *IL6*, *IL8*, and *TLR4* expression by 72%, 76%, and 46%, respectively. Since POPG is a surfactant-associated phospholipid, we tested whether POPG supplementation elicited a similar effect in this tissue model. To identify an effective POPG concentration, we compared 10, 25, and 50 μM POPG under LPS-stimulated conditions. The strongest and most consistent suppression of *IL6*, *IL8*, and *TLR4* expression was observed at 25 μM POPG (Figure S9A), which was therefore selected for subsequent analyses. Consistent with a suppressive effect, POPG reduced LPS-induced inflammatory gene expression in the *ex ovo* CAM model to a magnitude similar to VPA (Figure 5A; Figure S9B).

Histological evaluation further supported the transcriptional data (Figure 5B). While LPS-treated CAMs exhibited pronounced structural disruption, including epithelial thickening, marked edema, and extensive mesenchymal cellular infiltration, these pathological features were markedly reduced in CAMs pretreated with VPA or supplemented with POPG. In these samples, the chorionic epithelium appeared thinner and more continuous, mesenchymal cellularity was decreased, and overall tissue architecture was better preserved (Figure 5B).

Collectively, these results demonstrate that VPA pretreatment and POPG supplementation mitigate LPS-induced inflammatory gene expression and histological hallmarks of inflammation in the quail CAM model, extending the observations from cellular systems to a more physiologically relevant *in vivo* model.

## Discussion

VPA is a pleiotropic compound that affects mitochondrial metabolism, lipid homeostasis, and organelle function across diverse model systems.^36^^,^^37^^,^^46^^,^^47^ Here, we use A549 cells to show that pharmacological treatment with VPA and/or partial genetic loss of *PGS1* reshape mitochondrial PG homeostasis, cellular bioenergetics, and phospholipid secretion. These changes are accompanied by altered inflammatory readouts in epithelial cells, placing mitochondrial PG biosynthesis at a functional intersection between metabolism, lipid release, and inflammatory signaling.

In A549 cells, VPA reduced PGS1 activity while cellular PG levels increased (Figure 1). This apparent dissociation argues for homeostatic control of PG abundance and is consistent with regulatory feedback in PG/CL pathways reported in other systems.^37^^,^^48^ Changes in mitochondrial phospholipid composition are known to modify membrane properties and influence the activity and interactions of membrane-associated proteins, including respiratory chain complexes.^49^^,^^50^^,^^51^^,^^52^ Together, these observations support a broader role for PG homeostasis in coordinating mitochondrial function with cellular lipid metabolism.^36^^,^^48^^,^^53^^,^^54^ Consistent with the importance of PG homeostasis, *PGS1*^+/−^ cells exhibited a reduced overall cellular phospholipid pool, the relative distribution of major phospholipid classes remained largely preserved, with CL representing the only significantly altered class (Figure S2). These findings indicate that partial impairment of the PG/CL biosynthetic pathway predominantly affects phospholipid abundance rather than overall phospholipid composition.

Given that mitochondrial lipid metabolism contributes to cellular phospholipid homeostasis, we next asked whether VPA-induced changes in mitochondrial PG metabolism are reflected in the secretion of surfactant-associated phospholipids. Mass spectrometry confirmed that A549 cells secrete PG and PC species that differ from those found in native human ATII cells,^42^^,^^55^^,^^56^^,^^57^ consistent with earlier observations.^58^^,^^59^ VPA selectively altered this secretory output by increasing total PC secretion while remodeling the molecular species composition of PG without changing its overall abundance (Figure 2). These data show that pharmacological perturbation can shift the PG molecular-species profile in an alveolar epithelial model A549, thereby connecting mitochondrial lipid synthesis to changes in epithelial secretory composition. Notably, VPA increased the relative abundance of POPG, a PG species with established anti-inflammatory properties mediated by competition with LPS for binding to the CD14-MD2-TLR4 complex.^7^^,^^8^ This shift suggests a partial move toward a more “surfactant-like” and potentially immunomodulatory PG profile, even in a carcinoma-derived epithelial model with a non-physiological baseline lipidome.

In contrast, VPA-induced remodeling of surfactant-associated lipids and PG species was largely absent in *PGS1*^*+/−*^ cells (Figure 2; Figure S4). Despite near-complete restoration of intracellular phospholipid levels by therapeutic VPA (Figures 1B and 1C; Figure S2), the secreted phospholipid profile remained largely unchanged, indicating that recovery of the intracellular phospholipid pool alone is insufficient to normalize epithelial phospholipid secretion. Moreover, VPA normalized the amount of secreted PG in this background but failed to induce qualitative changes in PG molecular species (Figure 2C). These results indicate that intact mitochondrial PG biosynthetic capacity contributes to VPA-induced remodeling of the PG secretome. The distinction between PG quantity and molecular composition further suggests that decreased PGS1 activity may limit the availability of specific PG molecular species rather than simply determining total PG levels. In parallel, mitochondrial demand for PG to sustain CL synthesis and respiratory function, which may further restrict the PG pool available for surfactant secretion. In line with this interpretation, yeast studies have shown that mitochondrial PG differs in acyl composition from PG accumulating outside mitochondria when PG turnover is perturbed,^53^ supporting the concept that mitochondrial requirements can shape the molecular features of PG accessible to extra-mitochondrial pools.

Mitochondrial lipid homeostasis is a recognized regulator of inflammatory signaling,^22^^,^^23^^,^^25^ with CL influencing the stability of respiratory complex II and cytokine production.^24^ Since defects in CL synthesis often coincide with PG accumulation,^36^^,^^37^^,^^53^ the *PGS1*^*+/−*^ model allowed us to evaluate epithelial inflammatory responses under conditions of impaired mitochondrial phospholipid biosynthesis. Under these conditions, *PGS1*^+/−^ cells showed elevated basal *IL8* mRNA expression but a diminished transcriptional response to LPS, despite comparable IL-8 secretion and a modest increase in intracellular IL-8 protein levels (Figures 3C–3E; Figure S5). These findings indicate a dissociation between *IL8* transcript abundance and intracellular or extracellular IL-8 protein levels in *PGS1*^+/−^ cells. Such uncoupling is consistent with post-transcriptional regulation, including differences in mRNA stability and translation, as previously described under conditions of cellular stress and inflammatory activation.^60^^,^^61^ Although the mechanisms controlling *IL8* transcription, mRNA stability, and translation have been extensively studied,^62^^,^^63^^,^^64^ comparatively less is known about how IL-8 protein turnover and secretion contribute to the regulation of extracellular IL-8 protein levels. Interestingly, VPA enhanced LPS-induced *IL8* expression, cellular IL-8 protein levels, and IL-8 secretion while simultaneously restoring mitochondrial PG and CL levels in *PGS1*^+/−^ cells (Figures 1A–1F). Together, these findings support an association between mitochondrial phospholipid homeostasis and epithelial inflammatory responsiveness, although the mechanisms linking these processes remain to be established.

To test whether epithelial lipid remodeling influences macrophage activation, conditioned media from WT and *PGS1*^+/−^ A549 cells were applied to U937-derived macrophages and consistently reduced pro-inflammatory marker expression (Figures 4A and 4B). Comparing whole conditioned media with corresponding lipid extracts revealed that lipids partially reproduced this suppressive effect (Figure 4C; Figure S6A). Thus, secreted lipids contribute to macrophage modulation, but additional components of the conditioned medium likely participate. Importantly, lipid extracts from both epithelial backgrounds produced similar responses, suggesting that differences in PG molecular species alone are unlikely to be the dominant determinant of macrophage suppression in this experimental setup. This conclusion is further supported by the very low PG concentrations detected in conditioned media (∼10 ng/mL), which are several orders of magnitude below both the concentration of exogenously applied POPG (25 μM; ∼19 μg/mL) and PG concentrations in native pulmonary surfactant (∼5 mg/mL).^5^ Accordingly, the suppressive activity of conditioned media is unlikely to be explained by PG alone at the concentrations detected here and may instead reflect combined effects of multiple lipid classes and/or lipid-associated factors.

To address limitations of simplified *in vitro* systems and test tissue-level relevance, we used the CAM of Japanese quail embryos as an *in vivo* model. CAM is functionally and morphologically similar to the lung tissue and has been used as a model to study pulmonary health issues.^65^ In this system, both VPA and exogenously applied POPG significantly attenuated LPS-induced expression of *IL6*, *IL8*, and *TLR4* and preserved tissue architecture (Figure 5). While the CAM does not recapitulate pulmonary-specific structure or surfactant recycling, it provides a multicellular, vascularized inflammatory environment in which pharmacological modulation (VPA) and lipid supplementation (POPG) can be evaluated side-by-side. Similar protective effects of surfactant PG species have been reported *in vivo* in models of ARDS and viral pneumonia, supporting the translational relevance of targeting surfactant lipid composition in pulmonary inflammation.^9^^,^^10^^,^^11^

**Figure 6:**
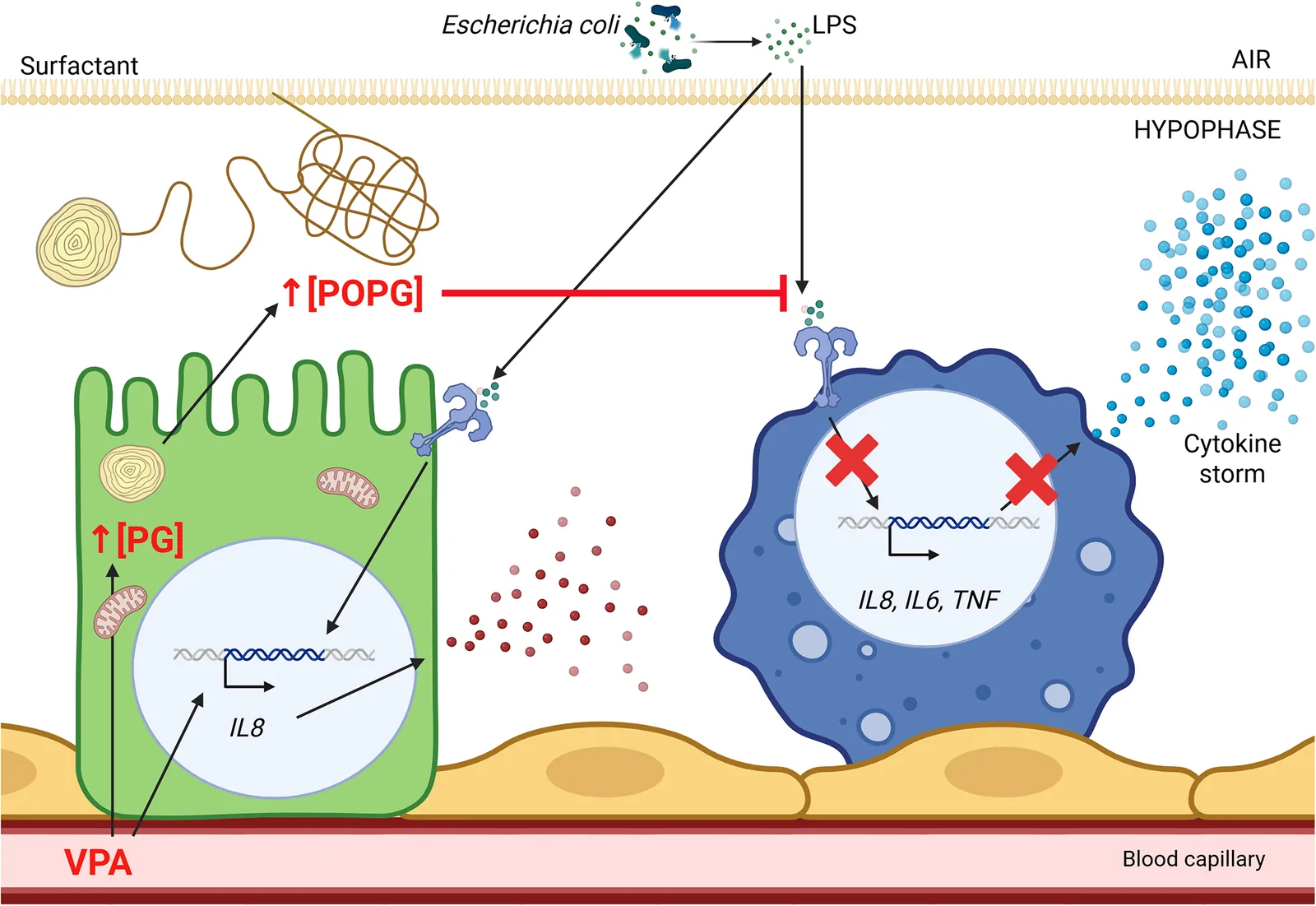
Proposed model of VPA-dependent modulation of epithelial-macrophage inflammatory signaling Using A549 cells as a model of ATII epithelial cells, our data suggest that inflammatory stimulation, further enhanced by VPA treatment, increases IL-8 production, thereby strengthening the chemotactic signal for monocyte recruitment to inflamed tissue. After recruitment, monocytes exposed to LPS differentiate toward a macrophage-like phenotype; however, in the presence of VPA, their production of key pro-inflammatory mediators is attenuated. This supports a model in which VPA promotes immune cell accumulation at inflammatory sites while simultaneously dampening macrophage inflammatory output, potentially shifting the local immune response toward a more controlled, regulated state.

In summary, our results highlight mitochondrial PG biosynthesis as a lever that influences epithelial metabolism, lipid secretion, and inflammatory signaling. Rather than acting as a uniformly anti-inflammatory agent, VPA exerted context-dependent effects: in alveolar epithelial cells, it increased inflammatory responsiveness (notably *IL8*), whereas in macrophage-like cells and in the CAM model, it attenuated LPS-driven inflammatory outputs (Figure 6). These context-dependent responses form the basis of our proposed model of VPA-mediated modulation of epithelial-immune signaling. Overall, our findings position mitochondrial lipid homeostasis and surfactant-associated phospholipids as tractable nodes for therapeutic modulation in inflammatory lung disease.

### Limitations of the study

Several limitations of this study should be acknowledged. We relied on carcinoma-derived epithelial cells and simplified immune models, and we did not resolve the intracellular mechanisms linking mitochondrial PG synthesis to lipid trafficking and secretion. Moreover, VPA exerts pleiotropic effects that cannot be fully attributed to mitochondrial lipid metabolism. Nonetheless, the combination of genetic, pharmacological, and lipid supplementation data supports a model in which mitochondrial lipid homeostasis modulates secretory composition and, in turn, inflammatory signaling across cell types and at the tissue level.

## Resource availability

### Lead contact

Further information and requests for resources should be directed to and will be fulfilled by the lead contact, Mária Balážová (maria.balazova@savba.sk).

### Materials availability

The *PGS1*^*+/−*^ A549 cell line generated in this study is available from the lead contact upon reasonable request.

### Data and code availability

• Lipid mass spectrometry data have been deposited at MassIVE and are publicly available under accession number MassIVE: MSV000101425 (https://doi.org/10.25345/C5DB7W44T).
• This study does not report original code.
• Any additional information required to reanalyze the data reported in this study is available from the lead contact upon request.

## Acknowledgments

This work was supported by the Scientific Grant Agency of the Ministry of Education, Science, Research and Sport of the Slovak Republic and the 10.13039/100010783Slovak Academy of Sciences (VEGA; grant numbers 2/0012/26, and 2/0081/25), the 10.13039/501100005357Slovak Research and Development Agency (APVV; contract numbers APVV-20-0129 and VV-MVP-24-0335), the 10.13039/100010783Slovak Academy of Sciences (grant SAS-NSTC/JRP/2023/773/LipAI), and the 10.13039/501100001824Grant Agency of the Czech Republic (GAČR; grant 26-22338S). Figure 6 was created using BioRender.com.

## Author contributions

D.I. performed experiments, analyzed data, and drafted the manuscript. P.S. generated the *PGS1*^+/−^ A549 cells and conducted related analyses. K.A. and F.D. performed mass spectrometry. M. Mariana, M. Mailinda, Z.I., and B.B. handled CAM experiments, including RT-PCR and histology. B. Miroslav conducted Seahorse metabolic assays. B.S. and F.I. measured secreted cytokines. H.H.Y.-H. and C.I. contributed to data analysis. F.D. financially supported the generation and characterization of the mutant cell line. B. Mária conceived and supervised the study, acquired funding, and oversaw methodology, visualization, validation, and manuscript preparation. All authors reviewed and approved the final manuscript.

## Declaration of interests

The authors declare no conflicts of interest.

## STAR★Methods

### Key resources table

**Table:** 

REAGENT or RESOURCE	SOURCE	IDENTIFIER
**Antibodies**		

Mouse OxPhos Human WB Antibody Cocktail	Invitrogen	Cat# 45-8099; RRID: AB_2533835
Goat anti-Mouse IgG (H + L), HRP-conjugated	Merck	Cat# A4416; RRID: AB_258167

**Chemicals, peptides, and recombinant proteins**		

Valproic acid sodium salt (VPA)	Sigma-Aldrich	Cat# P4543
Lipopolysaccharides from *Escherichia coli* O111:B4	Merck	Cat# L4130
Lipofectamine 2000	Invitrogen	Cat# 11668027
Phorbol 12-myristate 13-acetate (PMA)	Santa Cruz	Cat# sc-3576A
1-palmitoyl-2-oleoyl-*sn*-glycero-3-phospho-(1′-rac-glycerol) (POPG)	Avanti Polar Lipids	Cat# 840457P
Cardiolipin (CL)	Avanti Polar Lipids	Cat# 710335P
Dipalmitoyl phosphatidylglycerol (DPPG)	Avanti Polar Lipids	Cat# 840455P
Phosphatidic acid (PA)	Avanti Polar Lipids	Cat# 840101C
Phosphatidylcholine (PC)	Avanti Polar Lipids	Cat# 840051C
Phosphatidylethanolamine (PE)	Avanti Polar Lipids	Cat# 840021C
Phosphatidylinositol (PI)	Avanti Polar Lipids	Cat# 840042C
Phosphatidylserine (PS)	Avanti Polar Lipids	Cat# 870336C
Recombinant human IL-8	PeproTech	Cat# 200-08
SPLASH Lipidomix Mass Spec Standard	Avanti Polar Lipids	Cat# 330707
[^14^C]glycerol-3-phosphate	American Radiolabeled Chemicals	Cat# ARC 0316
CDP-diacylglycerol (CDP-DAG)	Avanti Polar Lipids	Cat# 870510C

**Critical commercial assays**		

GeneJET RNA Purification Kit	Thermo Scientific	Cat# K0732
Aurum Total RNA Mini Kit	Bio-Rad	Cat# 7326820
LunaScript RT SuperMix Kit	New England Biolabs	Cat# E3010L
FastStart Essential DNA Green Master	Roche	Cat# 06402712001
Human IL-6 ELISA kit	PeproTech	Cat# 900-T16
Human IL-8 ELISA kit	PeproTech	Cat# 900-T18
Human TNF-α ELISA kit	PeproTech	Cat# 900-T25
Bradford Protein Assay	Bio-Rad	Cat# 5000006
DC Protein Assay	Bio-Rad	Cat# 5000111
No-Stain Protein Labeling Reagent	Invitrogen	Cat# A44449
SuperSignal West Pico PLUS Chemiluminescent Substrate	Thermo Scientific	Cat# 34580
Seahorse XF Cell Mito Stress Test Kit	Agilent Technologies	Cat# 103015-100

**Deposited data**		

Raw and analyzed data	This paper; MassIVE	MassIVE: MSV000101425; https://doi.org/10.25345/C5DB7W44T

**Experimental models: Cell Lines**		

Human: A549 lung epithelial cells (WT)	ATCC	ATCC CCL-185
Human: A549 lung epithelial cells (*PGS1*^*+/−*^)	This paper	N/A
Human: U937 monocytic cell line	ATCC	ATCC CRL-1593.2

**Experimental models: Organisms/strains**		

Japanese quail (Coturnix japonica): *ex ovo* CAM model	N/A	N/A

**Oligonucleotides**		

Primers for RT-qPCR, see Table S1	This paper	N/A

**Recombinant DNA**		

pX330-U6-Chimeric_BB-CBh-hSpCas9	Addgene	Plasmid #42230

**Software and algorithms**		

Quantity One v4.5.2	Bio-Rad	N/A
Analyst	SCIEX	N/A
SCIEX OS v1.6.1	SCIEX	N/A
SigmaPlot 14	Inpixon	N/A
GraphPad Prism v10	GraphPad Software	N/A
IKOSA Prisma	Kolaido	N/A
BioRender	BioRender	https://www.biorender.com
CRISPOR	Concorded and Haeussler^66^	http://crispor.org

**Other**		

Seahorse XF Pro Analyzer	Agilent Technologies	N/A
ExionLC system	SCIEX	N/A
QTRAP 6500+ mass spectrometer	SCIEX	N/A
Acquity BEH C8 column, 2.1 × 100 mm, 1.7 μm	Waters	N/A
AriaMx Real-Time PCR System	Agilent Technologies	N/A
Silica Gel 60 TLC plates	Merck	Cat# 1.05553
Kapa 2000 optical microscope	Kvant	N/A
EOS 6D Mark II camera	Canon	N/A

### Experimental model and study participant details

#### WT and *PGS1*^*+/−*^ A549 cell culture

A549 human alveolar basal epithelial cells (WT and *PGS1*^+/−^), originally derived from a human lung adenocarcinoma of a female patient,^67^ were cultured in low-glucose Dulbecco’s Modified Eagle’s Medium (DMEM) supplemented with 10% fetal bovine serum (FBS) and 100 U/mL penicillin/streptomycin. Cells were maintained at 37°C in a humidified incubator with 5% CO_2_.

Unless stated otherwise, WT and *PGS1*^+/−^ cells were seeded at 2 × 10^5^ cells per 90 mm tissue-culture dish (2.5 × 10^4^ cells/mL in 8 mL medium) and maintained as static monolayers for 7 days with medium replacement every 2–3 days.^68^ Conditioned medium was collected after a 2-day incubation in serum-free medium.

#### U937 cell culture and differentiation

U937 human monocytic cells, originally derived from a male patient, were cultured in RPMI-1640 supplemented with 10% defined FBS and 100 U/mL penicillin/streptomycin at 37°C in 5% CO_2_. Differentiation was induced by seeding 8 × 10^6^ U937 cells per 90 mm dish (1 × 10^6^ cells/mL in 8 mL medium) and treating with PMA (10 ng/mL) for 48 h. Non-adherent cells were removed by PBS wash. Adherent cells were cultured in fresh medium for 24 h before stimulation.

#### Cell line authentication and mycoplasma testing

The A549 WT and U937 cell lines were obtained from ATCC (A549, CCL-185; U937, CRL-1593.2) and were not independently authenticated in our laboratory. The *PGS1*^*+/−*^ genotype of the A549 cells was confirmed by DNA sequencing of the genomic region encompassing the targeted *PGS1* variant. Cells were tested for mycoplasma contamination and were confirmed to be mycoplasma-free.

#### *Ex ovo* CAM model

The *ex ovo* Japanese quail (*Coturnix japonica*) CAM model was prepared in accordance with our previously reported methodology.^69^ Embryos were maintained at 37.5°C and 60–70% humidity. On embryonic day 8 (ED8), sterile silicone rings (⌀ 6 mm) were placed on the CAM to define the treatment area. The sex of the embryos was not determined; therefore, potential sex-related effects could not be evaluated.

#### Ethical statements

Experiments using the CAM of Japanese quail were conducted at or before ED10. According to EU Directive 2010/63/EU on the protection of animals used for scientific purposes, experiments performed on avian embryos prior to the final third of their development are not considered animal experimentation and therefore do not require institutional ethical approval or oversight. All procedures were carried out in accordance with applicable guidelines and regulations.

### Method details

#### Generation of a hypomorphic *PGS1* mutant cell line

To generate a hypomorphic *PGS1* mutant (*PGS1*^+/−^), we employed the CRISPR/Cas9 gene-editing system. The single-guide RNA (sgRNA) was designed using the CRISPOR design tool^66^ targeting Exon 2 of the *PGS1* gene as follows: sgRNA-F: 5′-caccg AGTCTCCAGTTCTCACGTTA-3′ and sgRNA-R: 5′-aaacTAACGTGAGAACTGGAGACT-3′. sgRNA oligonucleotides were annealed and cloned into the *Bbs*I-digested pX330-U6-Chimeric_BB-CBh-hSpCas9 plasmid (plasmid #42230, Addgene). A549 cells were transfected with 1 μg of pX330-sgRNA-PGS1 using Lipofectamine 2000 (#11668027, Invitrogen). After 48 h, transfected cells were seeded individually on 96-well plates. Single-cell clones were expanded, validated by PCR analysis using the following primers: PGS1_GT_F: 5′-AGCTGTTCCCCAGGTCAC-3′ and PGS1_GT_R: 5′-CCTCCCAAAGTACTGGCCA-3′, Sanger sequencing, and phenotype characterization. Loss of full-length *PGS1* transcript was assessed by qRT-PCR (primer sequences in Table S1).

#### Treatments, stimulation, and sample collection

For epithelial pretreatment, WT and *PGS1*^+/−^ cells were incubated with VPA (0, 0.06, or 0.6 mM) for 48 h. The selected VPA concentrations were based on the therapeutic serum range (0.346–0.693 mM),^70^ and our previous findings demonstrating biological activity at a subtherapeutic concentration.^36^^,^^37^ Cells were then stimulated with phospholipid vesicles (POPG; 10, 25, or 50 μM, corresponding to approximately 7.7, 19.3, and 38.5 μg/mL, respectively), LPS from *Escherichia coli* O111:B4 (Merck L4130, 1 μg/mL) (Figures S10A and S10B), or a premixed combination. Cells were harvested after 2 h, washed with PBS, and stored at −20°C for downstream analyses. Conditioned medium was collected for ELISA, lipid mass spectrometry analyses, and lipid extracts.

For macrophage-like assays, conditioned media were generated from WT and *PGS1*^+/−^ A549 cultures treated as indicated above. PMA-differentiated U937 cells were stimulated with LPS (0.1 μg/mL) (Figures S10C–S10E) in the presence of conditioned media and/or lipid extracts prepared from conditioned media. For exogenous phospholipid supplementation experiments, phospholipid vesicles were added at 25 μM or at the indicated concentrations for dose-response analyses (Figures S6B and S6C). Cells and medium were harvested after 6 h and stored at −20°C.

For CAM experiments, VPA (0, 0.06, or 0.6 mM in saline) was applied for 24 h. On ED9, LPS (100 μg/mL; 8 μg in 80 μL), POPG (25 μM; 1.54 μg in 80 μL), or their combination was added to the same area for 2 h for RT–PCR analysis (Figure S9F) and for 24 h for histology.

#### Lipid extraction from conditioned media

Lipids were extracted from 5 mL conditioned media of A549 cells using chloroform:methanol:HCl (60:30:0.2, v/v/v) for 1 h at 30°C. Phase separation was induced by the addition of 0.1 M MgCl_2_, followed by vortexing and incubation for 30 min. Samples were centrifuged (460 g, 10 min). The organic phase was collected, dried, and resuspended in culture medium. Lipid suspensions were sonicated to form vesicles and applied to U937 cells. Extracts were normalized to the original conditioned-medium volume, ensuring that lipid extracts derived from equivalent volumes of conditioned medium were applied across all experimental groups

#### Protein quantification and normalization

Total cellular protein (Bradford assay; Bio-Rad) was recorded per dish to document culture expansion and was used for normalization where indicated. For Seahorse XF measurements, protein content was determined using the DC Protein Assay (Bio-Rad).

#### RNA extraction and quantitative real-time PCR

Total RNA from human cells was isolated using the GeneJET RNA Purification Kit (Thermo Scientific), and total quail RNA from CAM tissue within the ring area was isolated using the AURUM total RNA mini kit (Bio-Rad). One microgram of RNA per sample was reverse-transcribed with the LunaScript RT SuperMix Kit (New England Biolabs). RT-PCR was performed on an AriaMx Real-Time PCR System (Agilent Technologies) using the FastStart Essential DNA Green Master (Roche). Gene expression levels were normalized to *hACTB* and *hGAPDH* for human samples and to *qGAPDH* for CAM tissue. Reported values represent relative expression calculated using the ΔΔCt method. Primer sequences are listed in Table S1.

#### Enzyme-linked immunosorbent assays (ELISA)

Concentrations of IL-6, TNF-α, and IL-8 in cell culture supernatants were measured using ELISA kits (PeproTech) following the manufacturer’s instructions. To determine intracellular IL-8 levels, cells were washed twice with PBS, homogenized using a Dounce homogenizer, and equal amounts of total protein were analyzed using ELISA. Absorbance was measured at the specified wavelength. Cytokine concentrations were determined from standard curves generated with kit-provided standards.

#### CAM histology

On ED9, CAM tissue was excised and fixed in 4% paraformaldehyde. The samples were dehydrated through a graded ethanol series and embedded in paraffin. After 24 h, tissues were overlaid with Paraplast Plus (Sigma-Aldrich) and embedded in casting molds. Serial sections (5 μm) were cut from three distinct regions of each sample using a microtome (Auxilab S.L.) and stained with hematoxylin and eosin (Bamed). Histological sections were imaged using an optical microscope Kapa 2000 (Kvant) at 8×, 20×, and 40× magnification with a Canon EOS 6D Mark II camera.

#### PGS1 activity assay

PGS1 enzymatic activity was assessed by measuring the incorporation of radiolabeled substrate into chloroform-soluble phospholipids as described previously.^37^ Whole-cell homogenates (75 μg protein) were incubated for 60 min at 37°C in 50 mM MES-HCl (pH 7.0), 0.1 mM MnCl_2_, 0.083 mM CDP-DAG, 1 mM Triton X-100, and 0.02 mM [^14^C]glycerol-3-phosphate (40,000 DPM) in 120 μL. The reaction was terminated with chloroform:methanol:HCl (100:100:0.6, v/v/v), followed by phase partitioning with water. The organic layer was collected, evaporated, and the lipid products were resolved on Silica Gel 60 TLC plates using chloroform:methanol:acetic acid:water (75:45:3:1, v/v/v/v) as the developing solvent. Radiolabeled PG was visualized by phosphorimaging and quantified using Quantity One software (Bio-Rad).

#### Determination of inorganic phosphate in phospholipids

Phospholipids were extracted from 1.8 mg of cell proteins with chloroform:methanol:HCl (60:30:0.2, v/v/v) for 1 h at 30°C, followed by 0.1 M MgCl_2_ addition, vortexing, 30 min incubation, and centrifugation (460 g, 10 min). Lipids were separated by 2D-TLC (chloroform:methanol:ammonia:water 66:27:3:0.8 and chloroform:methanol:acetic acid 65:25:8, v/v/v), visualized with iodine vapors, scraped for analysis as described.^54^ Briefly, scraped silica gel was mineralized with H_2_SO_4_:HClO_4_ (9:1, v/v) at 180 °C for 30 min, incubated with ANSA–molybdate reagent at 105°C for 30 min, and the absorbance was measured at 830 nm. Phosphate content was determined from a K_2_HPO_4_ standard curve and normalized to total protein (μg Pi/mg protein).

#### Seahorse mitochondrial assay

WT and *PGS1*^+/−^ A549 cells were seeded at 4 × 10^3^ cells per well in a Seahorse XF Pro M microplate and cultured for 7 days. Medium was replaced with Seahorse XF DMEM (pH 7.4) containing 10 mM glucose, 2 mM L-glutamine, and 1 mM pyruvate, and cells were equilibrated at 37°C for 1 h. Oxygen consumption rate and ECAR (extracellular acidification rate) were measured using a Seahorse XF Pro Analyzer. Mitochondrial function was probed with oligomycin (1.25 μM), FCCP (1 μM), and rotenone + antimycin A (1 μM + 1.25 μM). Data were normalized to protein content measured in each well.

#### Western blot analysis

Proteins were separated on 12% SDS-PAGE gels and transferred to nitrocellulose membranes. Membranes were blocked for 1 h in 5% milk in TBS (50 mM Tris-HCl, pH 8.0; 150 mM NaCl) and incubated overnight at 4°C with mouse OxPhos Human WB Antibody Cocktail (1:500; Invitrogen). HRP-conjugated secondary antibodies (anti-mouse, 1:30,000; Merck) were detected using SuperSignal West Pico PLUS Chemiluminescent Substrate (Thermo Scientific). Signal intensity was normalized to total protein using No-Stain Protein Labeling Reagent (Invitrogen).

#### Targeted lipidomics analysis

WT and *PGS1*^+/−^ A549 cells were cultured in DMEM for 7 days. Two days prior to medium collection, the medium containing 10% FBS was replaced with FBS-free medium. The culture medium was collected, centrifuged for 5 min at 460 g, and stored at −20°C until lipid extraction. Lipids were extracted from cell culture medium using a two-phase MTBE/methanol protocol described in.^71^ Samples were thawed on ice, vortexed, and 100 μL was transferred to 2 mL tubes. SPLASH Lipidomix internal standard (Avanti Polar Lipids) (10 μL) was added, followed by vortexing and 15 min incubation on ice. Blank samples were prepared in parallel using ultrapure water without internal standards. Cold methanol (375 μL) and MTBE (1,250 μL) were added sequentially with vortexing, and samples were incubated for 1 h at 4°C with orbital shaking (32 rpm). Phase separation was induced by adding 375 μL H_2_O, followed by 10 min incubation at 4°C. After centrifugation (10 min, 4°C, 1000 g), 1 mL of the upper organic phase was collected and evaporated under vacuum at room temperature (70 mbar). Dried extracts were stored at −80°C until analysis. Prior to LC-MS based lipidomic analysis, extracts were reconstituted in 100 μL isopropanol and vortexed, and 10 μL aliquots of each sample were pooled to generate a quality control sample (QC). Lipids were analyzed using a previously described targeted lipidomics LC-MS/MS method, with chromatographic and mass spectrometric conditions as reported earlier.^72^ The liquid chromatography ExionLC system (Sciex) with Acquity BEH C8 2.1 mm, 100 mm, 1.7 μm column (Waters) coupled to triple quadrupole mass spectrometer Sciex QTrap 6500+ (Sciex) was used. Data acquisition and processing were performed using Analyst (Sciex) and SCIEX OS software (Sciex, version 1.6.1), respectively. Results were presented as peak areas and normalized to cellular protein content and culture medium volume.

### Quantification and statistical analysis

Statistical analysis was performed in SigmaPlot 14 (Inpixon). Normality of data distributions was assessed using the D’Agostino–Pearson test. For comparisons among multiple groups, one-way ANOVA was used, followed by the Holm–Šidák multiple-comparisons procedure. Planned comparisons were performed as indicated in the figure legends. Unless stated otherwise, *n* denotes independent biological replicates (independently seeded cultures processed on different days). Graphs were prepared in GraphPad Prism 10 (GraphPad Software, San Diego, CA) and show mean ± SD. *p* values <0.05 were considered statistically significant.
