# Supplementary material for: Valproic acid remodels phosphatidylglycerol homeostasis and epithelial-macrophage crosstalk in inflammatory signaling
Source: iScience. 2026 Sep 4;29(9):117369. doi: 10.1016/j.isci.2026.117369 (PMC13571955; doi:10.1016/j.isci.2026.117369)
Supplement: Supplementary file 1 [file mmc1.pdf]

iScience, Volume *29*

Supplemental information

Valproic acid remodels phosphatidylglycerol homeostasis and epithelial-macrophage crosstalk in inflammatory signaling

Ivana Ďurišová, Mariana Máčajová, Ingrid Zriniová, Ivana Fábryová, Mailinda Meta, Lenka Bábelová, Boris Bilčík, Aleš Kvasnička, Miroslav Baláž, Soňa Bernátová, Silvia Petrezsélyová, Dominik Filipp, Yuan-Hao Hsu, David Friedecký, Ivan Čavarga, and Mária Balážová

SUPPLEMENTAL TABLE AND FIGURES

Table S1. List of primers (h = human, q = quail).

Name of primer	Sequence	Source
hACT_for	ATTGGCAATGAGCGGTTCCG	1
hACT_rev	AGGGCAGTGATCTCCTTCTG	
hGAPDH_for	CACCGTCAAGGCTGAGAAC	www.origene.com
hGAPDH_rev	GAGGGATCTCGCTCCTGG	
hIL8_for	ACATACTCCAAACCTTTCCACCC	2
hIL8_rev	CAACCCTCTGCACCCAGTTTTC	
hIL6_for	CCCACACAGACAGCCACTCA	This study
hIL6_rev	GTGCCTCTTTGCTGCTTTCAC	
hTNF_for	CTCTTCTGCCTGCTGCACTTTG	www.origene.com
hTNF_rev	ATGGGCTACAGGCTTGTCACTC	
hPGS1_e2_F	TTCTCACGTTAGGGTGCTTTC	This study
hPGS1_e3_R	TCCTGTTCCAAAGGACCTGT	
qGAPDH_for	TGATGCTCCCATGTTCGTGA	3
qGAPDH_rev	TAAGACCCTCCACGATGCC	
qIL8_for	CTGAGGTGCCAGTGCATTAG	4
qIL8_rev	AGCACACCTCTCTTCCATCC	
qIL6_for	GGTGATAAATCCCGATGAAGT	3
qIL6_rev	TCTCCATAAACGAAGTAAAGTCTC	
qTLR4_for	ATGTCCTCTTGCCATCCAA	3
qTLR4_rev	TCTCCCCTTTCTGCAGAGTG	

1. Kaminski, W.E., Jendraschak, E., Baumann, K., Kiefl, R., Fischer, S., Marcus, A.J., Broekman, M.J., and von Schacky, C. (1996). Human mononuclear cells express 12-LX: coordinated mRNA regulation with 5-LX and FLAP genes. Blood *87*, 331–340.

2. Eken, C., Martin, P.J., Sadallah, S., Treves, S., Schaller, M., and Schifferli, J.A. (2010). Ectosomes released by polymorphonuclear neutrophils induce a MerTK-dependent anti-inflammatory pathway in macrophages. J. Biol. Chem. *285*, 39914–39921. https://doi.org/10.1074/jbc.M110.126748.

3. Pu, S., Usuda, K., Nagaoka, K., Gore, A., Crews, D., and Watanabe, G. (2020). The relation between liver damage and reproduction in female Japanese quail (*Coturnix japonica*) exposed to high ambient temperature. Poult. Sci. *99*, 4586–4597. https://doi.org/10.1016/j.psj.2020.05.025.

4. Uno, Y., Usui, T., Fujimoto, Y., Ito, T., and Yamaguchi, T. (2012). Quantification of interferon, interleukin, and Toll-like receptor 7 mRNA in quail splenocytes using real-time PCR. Poult. Sci. *91*, 2496–2501. https://doi.org/10.3382/ps.2012-02283.

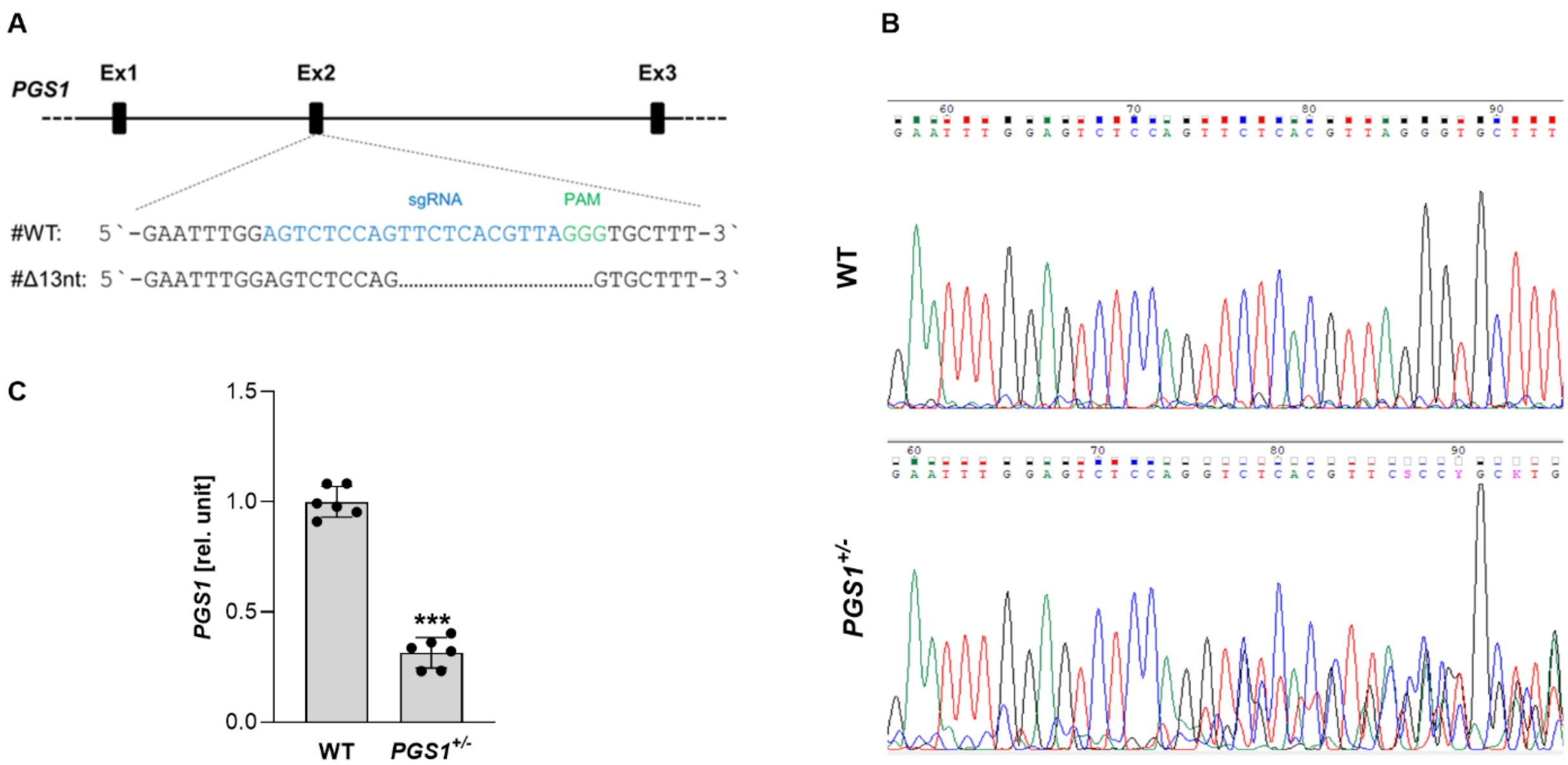

Figure S1. Generation of a heterozygous *PGS1* mutant in the A549 cell line by the CRISPR/Cas9 system.

(A) The sgRNA sequence (blue) targeting Exon 2 in the *PGS1* gene. GGG (green) is the PAM sequence. A mono-allelic modification was present in the selected clone, represented by the dashed line (deletion of 13 nucleotides). (B) Sanger sequencing results showing a 13 nt deletion in one allele. (C) *PGS1* mRNA levels in WT and *PGS1*$^{+/-}$ cells determined by RT–qPCR using two primers, of which one recognizes deleted exon 2 region and the other one binds to exon 3. The analysis was done using ΔΔCt method and data were normalized to *ACTB* and *GAPDH* and are shown relative to WT. Data are shown as mean ± SD from six independent experiments (individual points shown). Statistical significance was assessed using one-way ANOVA followed by the Holm–Šidák multiple-comparisons test. ***$p < 0.001$

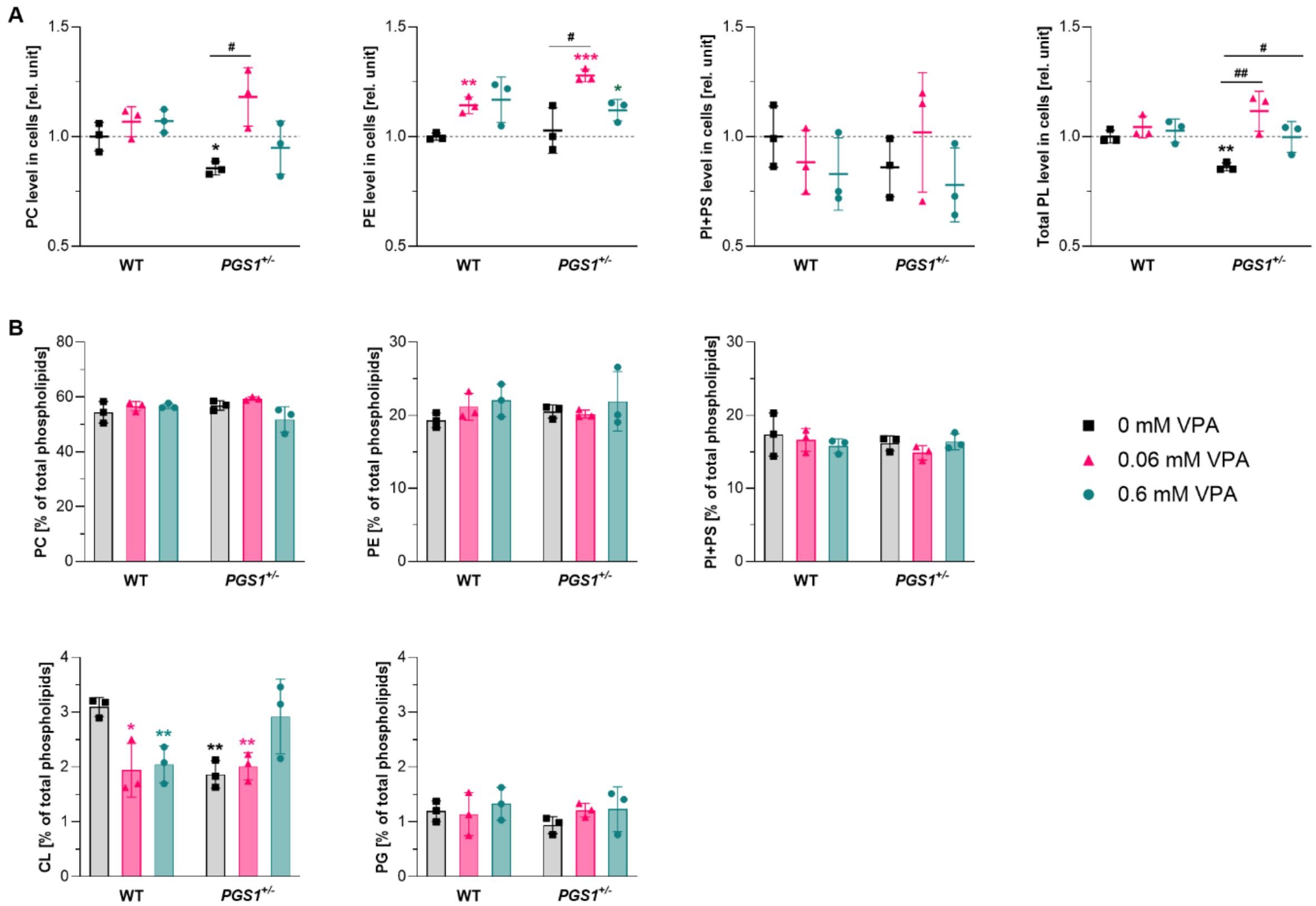

Figure S2. VPA restores phospholipid content in *PGS1*$^{+/-}$ cells without markedly altering phospholipid class distribution.

WT and *PGS1*$^{+/-}$ A549 cells were cultured in DMEM for 5 days and subsequently treated for an additional 2 days with 0, 0.06, or 0.6 mM VPA in DMEM lacking FBS. (A) Total phospholipid content and individual phospholipid classes (PC, PE, PI+PS, and CL) in WT and PGS1$^{+/-}$ A549 cells. Phospholipid amounts are expressed relative to total cellular protein. Data are normalized to untreated WT cells (0 mM VPA). (B) Relative distribution of phospholipid classes, expressed as the percentage of total phospholipids. Data from A and B represent mean ± SD from three independent experiments (individual points shown). Statistical significance was assessed using one-way ANOVA followed by the Holm–Šidák multiple-comparisons test. Asterisks indicate significant differences relative to untreated WT cells; hashtags denote significant differences among VPA concentrations within the same cell line. *$p < 0.05$; **$p < 0.01$; ***$p < 0.001$; $^{\#}p < 0.05$; $^{\#\#}p < 0.01$. CL, cardiolipin; PC, phosphatidylcholine; PE, phosphatidylethanolamine; PG, phosphatidylglycerol; PI, phosphatidylinositol; PS, phosphatidylserine; VPA, valproic acid; WT, wild type.

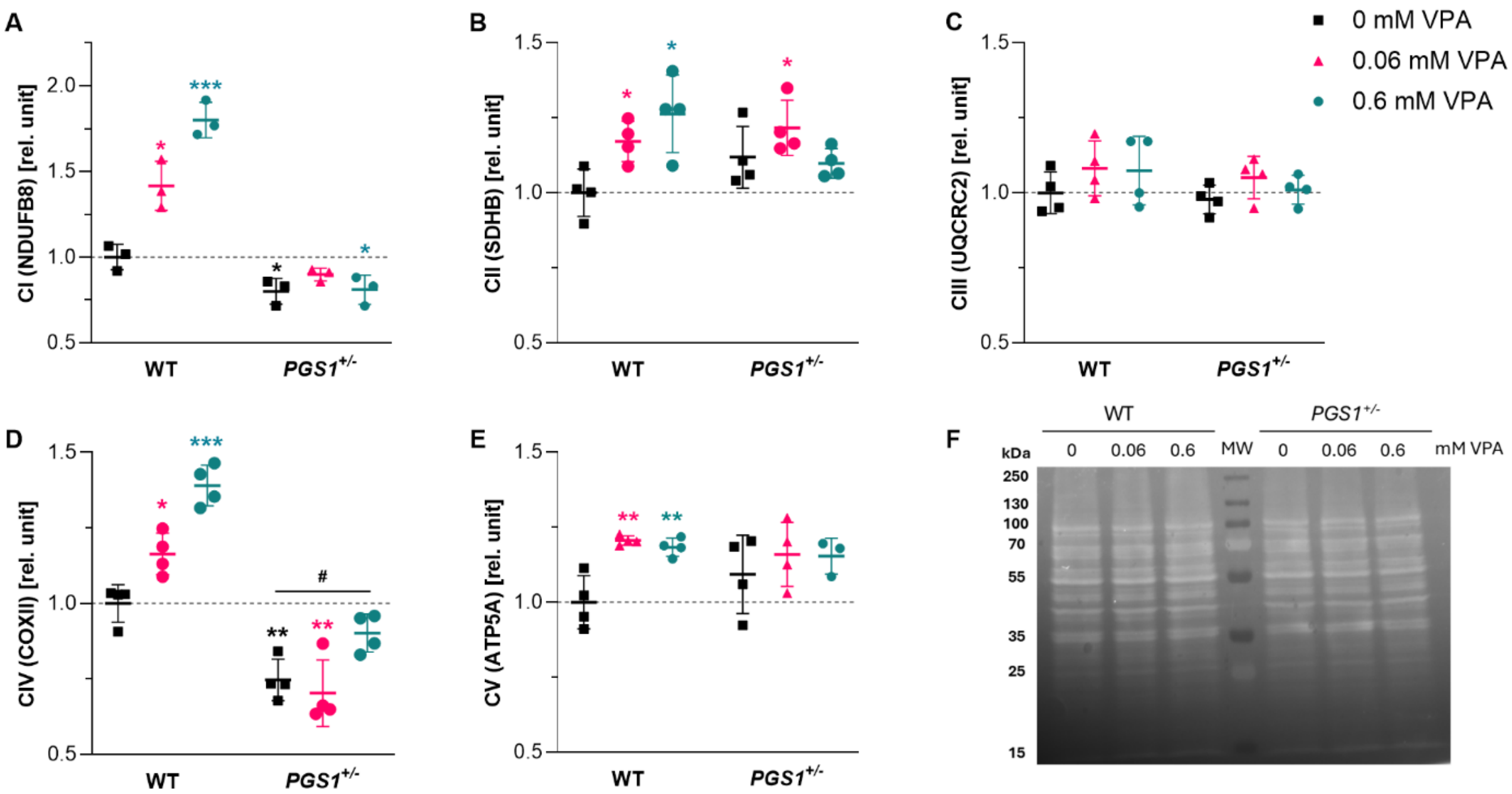

Figure S3. PG biosynthesis and VPA treatment alter respiratory chain protein levels

WT and *PGS1*$^{+/-}$ A549 cells were cultured in DMEM for 5 days and subsequently treated for an additional 2 days with 0, 0.06, or 0.6 mM VPA in DMEM lacking FBS. Western blot analysis was performed as described in Methods using the mouse OxPhos Human WB Antibody Cocktail. Signal intensity was normalized to total protein using the No-Stain™ Protein Labeling Reagent (Invitrogen). (A–E) Quantification of individual respiratory chain complex subunits and ATP synthase during the cultivation period: NDUFB8 (Complex I, CI), SDHB (Complex II, CII), UQCRC2 (Complex III, CIII), COXII (Complex IV, CIV), and ATP5A (ATP synthase, CV). Data are normalized to untreated WT cells (0 mM VPA) and represent mean ± SD from at least three independent experiments (individual data points shown). Statistical significance was assessed using one-way ANOVA followed by the Holm–Šidák multiple-comparisons test. Asterisks indicate significant differences relative to untreated WT cells; hashtags indicate significant differences among VPA concentrations within the same cell line. (F) Representative total protein staining used for normalization. *$p < 0.05$; **$p < 0.01$; ***$p < 0.001$; $^{\#}p < 0.05$. MW, molecular weight standard; VPA, valproic acid.

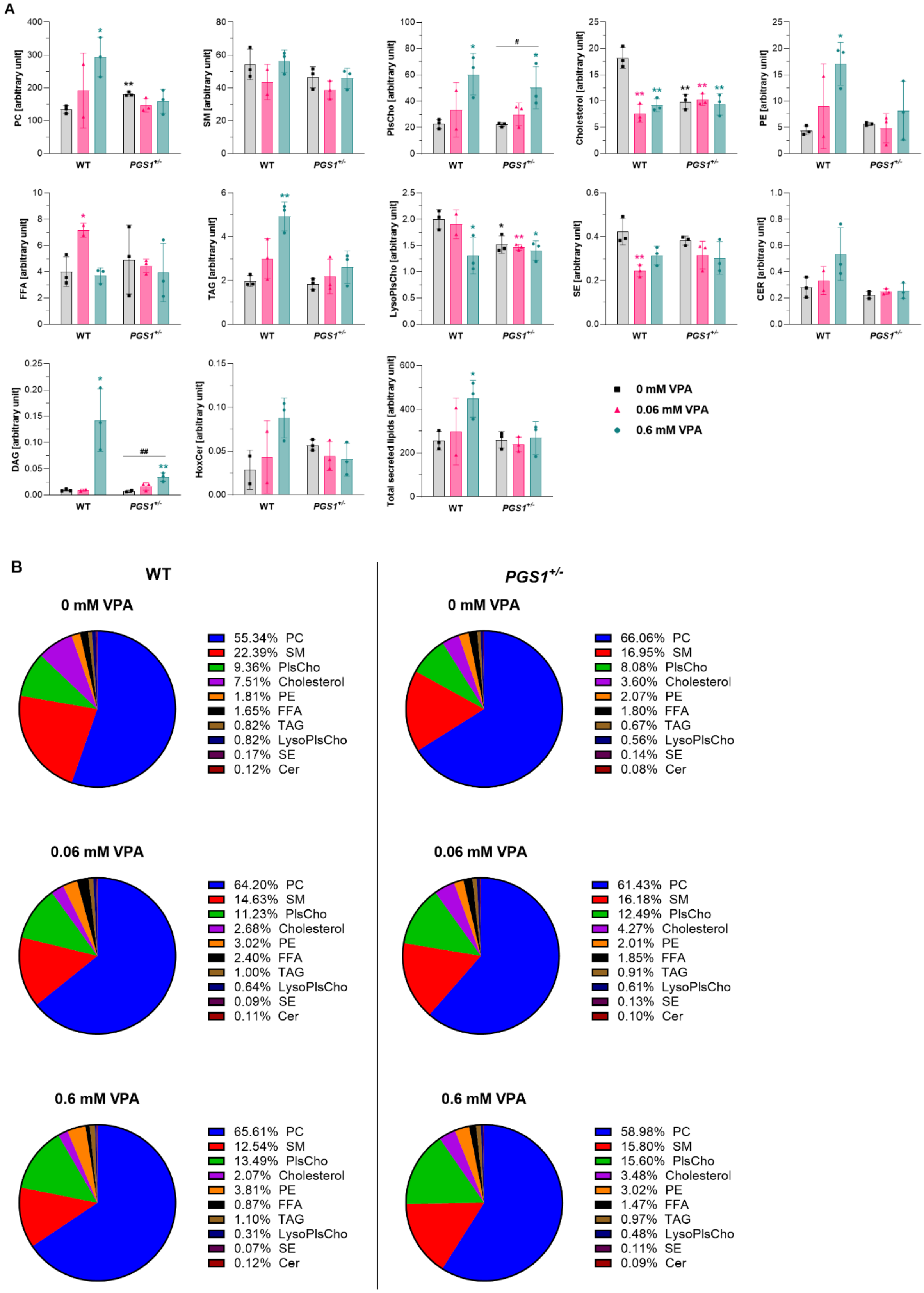
A
PC [arbitrary unit]
SM [arbitrary unit]
PlsCho [arbitrary unit]
Cholesterol [arbitrary unit]
PE [arbitrary unit]
FFA [arbitrary unit]
TAG [arbitrary unit]
LysoPlsCho [arbitrary unit]
SE [arbitrary unit]
CER [arbitrary unit]
DAG [arbitrary unit]
HoxCer [arbitrary unit]
Total secreted lipids [arbitrary unit]
WT
PGS1+/-
0 mM VPA
0.06 mM VPA
0.6 mM VPA
B
WT
PGS1+/-
0 mM VPA
55.34% PC
22.39% SM
9.36% PlsCho
7.51% Cholesterol
1.81% PE
1.65% FFA
0.82% TAG
0.82% LysoPlsCho
0.17% SE
0.12% Cer
0 mM VPA
66.06% PC
16.95% SM
8.08% PlsCho
3.60% Cholesterol
2.07% PE
1.80% FFA
0.67% TAG
0.56% LysoPlsCho
0.14% SE
0.08% Cer
0.06 mM VPA
64.20% PC
14.63% SM
11.23% PlsCho
2.68% Cholesterol
3.02% PE
2.40% FFA
1.00% TAG
0.64% LysoPlsCho
0.09% SE
0.11% Cer
0.06 mM VPA
61.43% PC
16.18% SM
12.49% PlsCho
4.27% Cholesterol
2.01% PE
1.85% FFA
0.91% TAG
0.61% LysoPlsCho
0.13% SE
0.10% Cer
0.6 mM VPA
65.61% PC
12.54% SM
13.49% PlsCho
2.07% Cholesterol
3.81% PE
0.87% FFA
1.10% TAG
0.31% LysoPlsCho
0.07% SE
0.12% Cer
0.6 mM VPA
58.98% PC
15.80% SM
15.60% PlsCho
3.48% Cholesterol
3.02% PE
1.47% FFA
0.97% TAG
0.48% LysoPlsCho
0.11% SE
0.09% Cer

Figure S4. Lipid secretion is affected by PG biosynthesis and VPA treatment

A549 (WT) and *PGS1*$^{+/-}$ cells were cultured in DMEM for 5 days and subsequently treated for an additional 2 days with 0, 0.06, or 0.6 mM VPA in DMEM lacking FBS. Culture supernatants were collected, centrifuged to clear, and analyzed by mass spectrometry to determine their lipid composition. (A) Levels of selected lipid species. (B) Ratios of selected lipid species with relative abundance higher than 0.1%. Data represent mean ± SD from three independent experiments (individual data points shown). Statistical significance was assessed using one-way ANOVA followed by the Holm–Šidák multiple-comparisons test. *p < 0.05; **p < 0.01; $^{\#}$p < 0.05; $^{\#\#}$p < 0.01. Cer, Ceramide; DAG, Diacylglycerol; FFA, Free fatty acids; HoxCer, Hydroxyceramide; LysoplsCho, Lysophosphatidylcholine; PC, Phosphatidylcholine; PE, Phosphatidylethanolamine; PlsCho, Plasmenyl phosphatidylcholine; PlsE, Plasmenyl phosphatidylethanolamine; SM, Sphingomyelin; SE, Sterol ester; TAG, Triacylglycerol; VPA, valproic acid

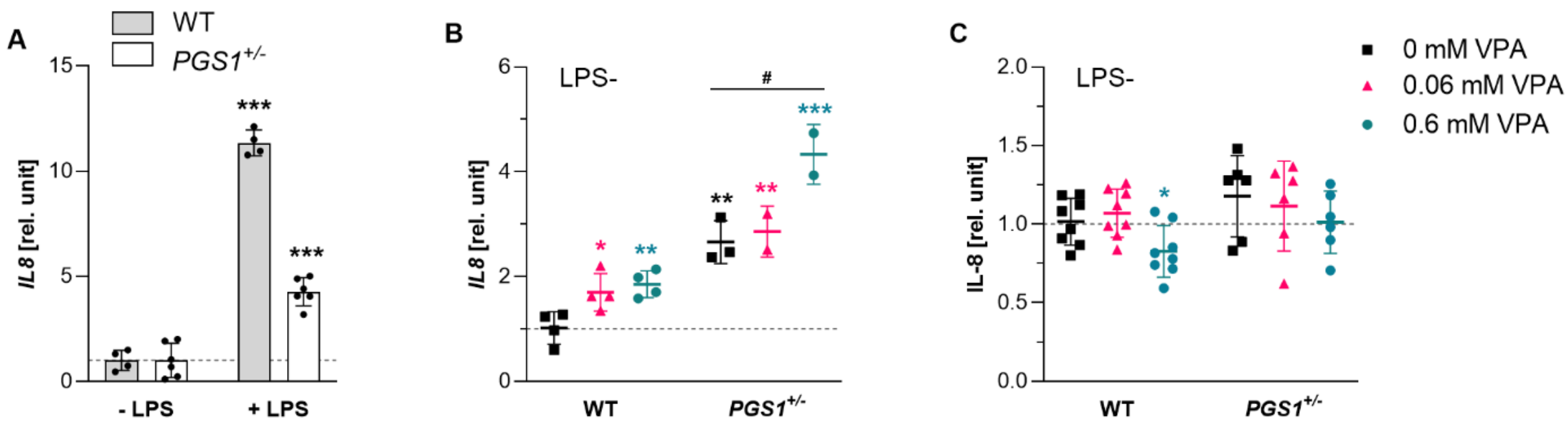

Figure S5. Expression of *IL8* in A549 epithelial cells is affected by PG biosynthesis WT and *PGS1*$^{+/-}$ A549 cells were cultured in DMEM for 5 days and subsequently treated for an additional 2 days with 0, 0.06, or 0.6 mM VPA in DMEM lacking FBS. (A) Relative mRNA levels of *IL8* under basal conditions and after induction of inflammation by treatment with 1 µg/mL LPS for 2 h. Data are normalized to WT or *PGS1*$^{+/-}$ cells without LPS treatment (−LPS) and represent mean ± SD from at least four independent experiments (individual points shown). Statistical significance was assessed using one-way ANOVA followed by the Holm–Šidák multiple-comparisons test. Asterisks indicate significant differences relative to untreated WT and LPS-treated WT cells, or between untreated *PGS1*$^{+/-}$ and LPS-treated *PGS1*$^{+/-}$ cells. (B) Relative mRNA level of *IL8* in cells and (C) protein level of IL-8 in culture media of cells pretreated with VPA in the absence of LPS. Data in (B) and (C) are normalized to untreated WT cells (0 mM VPA) and represent mean ± SD from at least two independent experiments (individual points shown). Statistical significance was assessed using one-way ANOVA followed by the Holm–Šidák multiple-comparisons test. Asterisks indicate significant differences relative to untreated WT cells; hashtags denote significant differences among VPA concentrations within the same cell line. *$p < 0.05$; **$p < 0.01$; ***$p < 0.001$; $^{\#\#}p < 0.01$; $^{\#\#\#}p < 0.001$. LPS, lipopolysaccharide; VPA, valproic acid

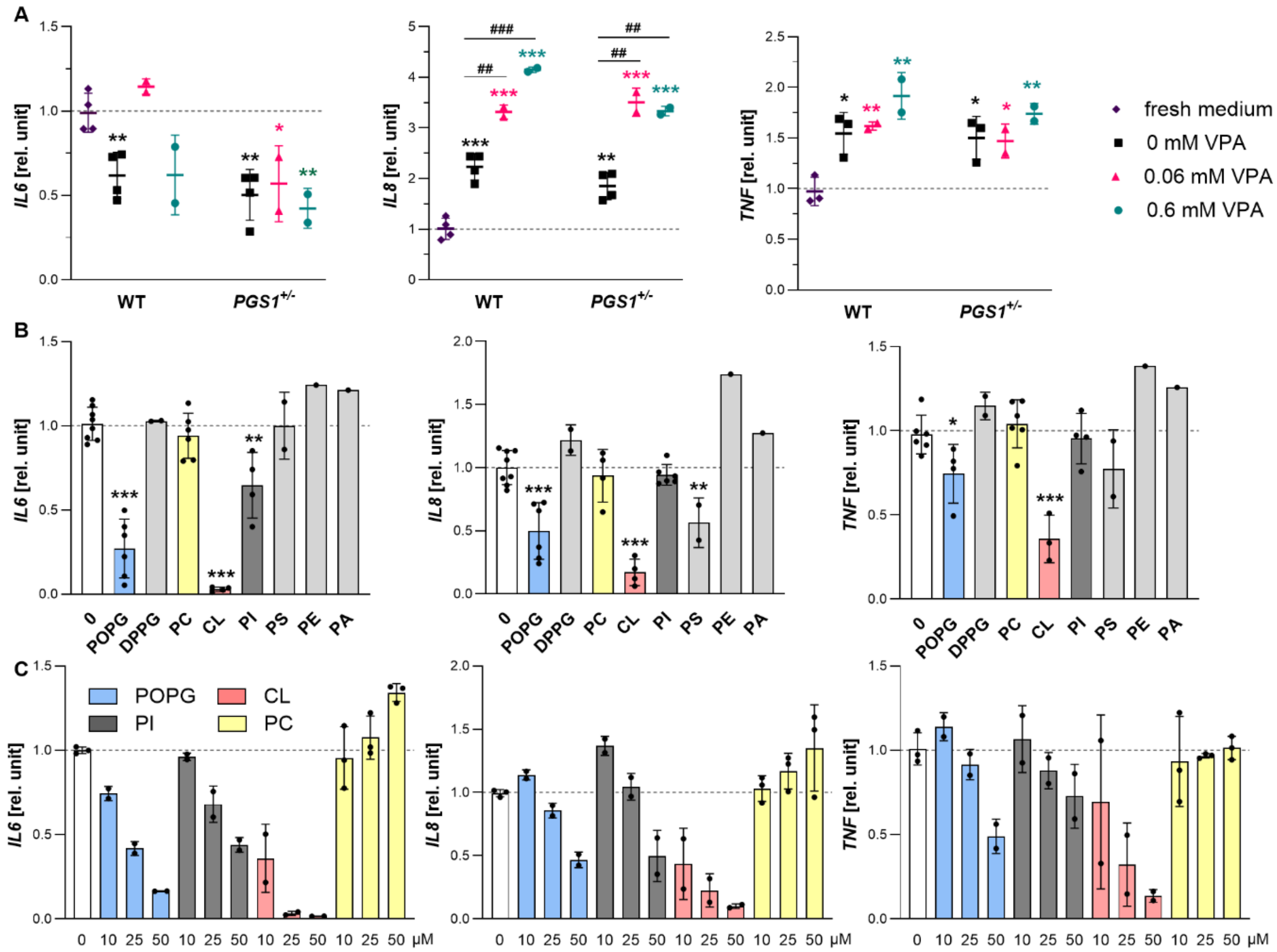

Figure S6. External addition of lipids affects the inflammatory response in U937 macrophages

Differentiation of U937 cells into macrophages was induced by seeding 8×10⁶ cells per 90 mm dish (1×10⁶ cells/mL in 8 mL) and treating them with PMA (10 ng/mL) for 48 hours. Non-adherent cells were removed by washing with PBS, and adherent cells were cultured in fresh medium for an additional 24 hours. Inflammation was induced in all samples by treatment with 0.1 μg/mL LPS for 6 hours. (A) Relative mRNA levels of *IL6*, *IL8*, and *TNF* in U937 cells stimulated with LPS in the presence of lipids extracted from conditioned media of WT and *PGS1*$^{+/-}$ A549 cells treated with 0, 0.06, or 0.6 mM VPA for 48 hours. Data represent mean ± SD from at least two independent experiments (individual points shown) and are normalized to U937 cells cultured in fresh medium. Statistical significance was assessed using one-way ANOVA followed by the Holm–Šidák multiple-comparisons test. Asterisks indicate significant differences relative to U937 cells in fresh medium; hashtags denote significant differences among VPA concentrations within the same conditioned medium. (B) Relative mRNA levels of *IL6*, *IL8*, and *TNF* in U937 cells stimulated with LPS in the presence of externally added phospholipids at a concentration of 25 μM. (C) Relative mRNA levels of *IL6*, *IL8*, and *TNF* in U937 cells stimulated with LPS in the presence of different concentrations of selected lipids, demonstrating dose-dependent effects on inflammation. Data represent mean ± SD from at least two independent experiments (individual points shown) and are normalized to U937 cells treated

without added lipids; in several cases, measurements were performed once to demonstrate the absence of an effect and are shown without statistical evaluation. Statistical significance was assessed using one-way ANOVA followed by the Holm–Šidák multiple-comparisons test. Asterisks indicate significant differences relative to U937 cells without externally added lipids. $^{*}p < 0.05$; $^{**}p < 0.01$; $^{***}p < 0.001$; $^{\#\#}p < 0.01$; $^{\#\#\#}p < 0.001$. CL, cardiolipin; DPPG, dipalmitoyl phosphatidylglycerol; PA, phosphatidic acid; PC, phosphatidylcholine; PE, phosphatidylethanolamine; PI, phosphatidylinositol; POPG, palmitoyl-oleoyl phosphatidylglycerol; PS, phosphatidylserine; VPA, valproic acid.

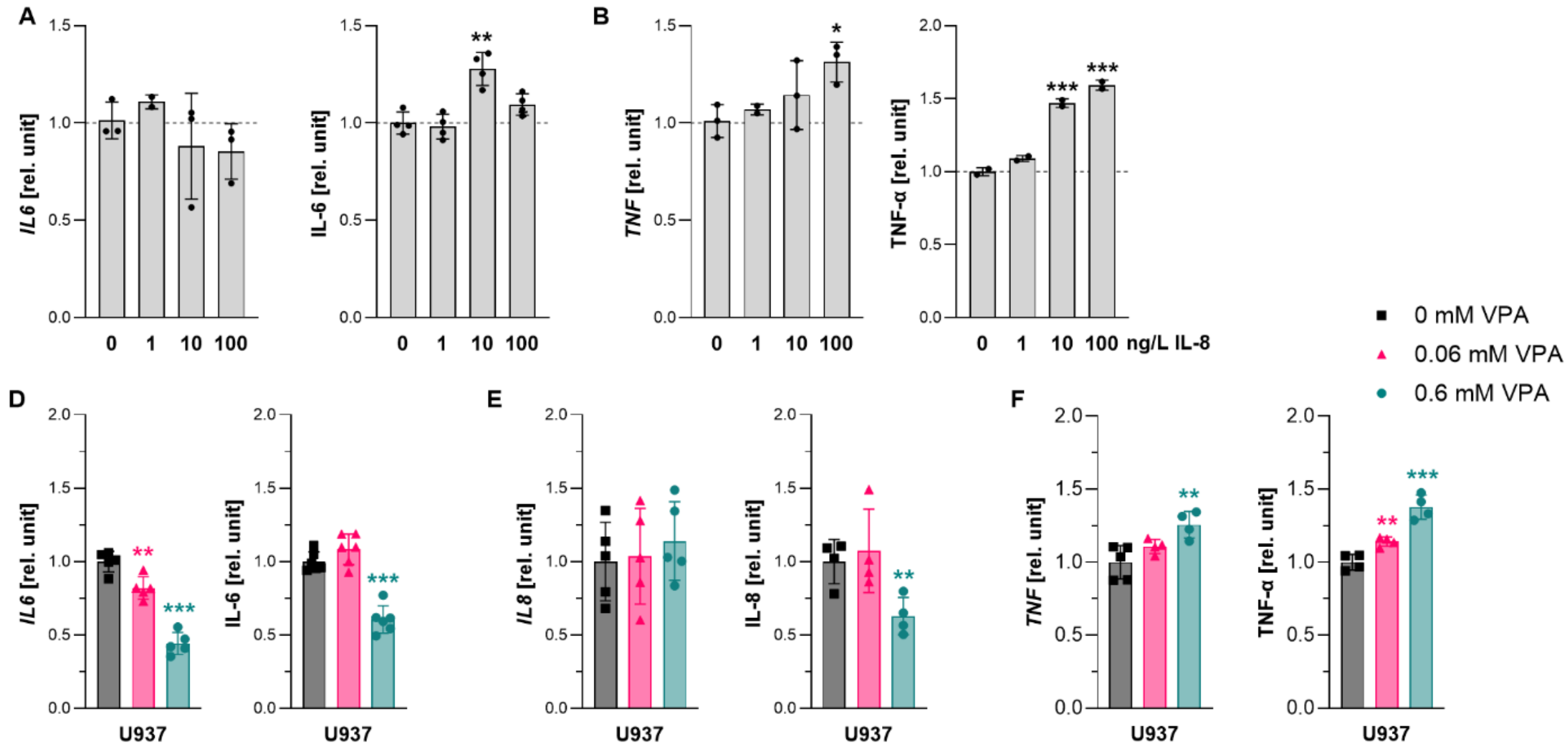

Figure S7. Effect of externally added IL-8 and VPA treatment on suppression of inflammation in U937 macrophages

Differentiation of U937 cells into macrophages was induced by seeding cells at 1 × 10^6 cells/mL and treating them with PMA (10 ng/mL) for 48 hours. Non-adherent cells were removed by washing with PBS, and adherent cells were cultured in fresh medium for an additional 24 hours. Inflammation was induced in all samples by treatment with 0.1 µg/mL LPS for 6 hours. (A) Relative mRNA levels of *IL6* in cells (left) and protein level of IL-6 in culture medium (right) of U937 cells after addition of different concentrations of IL-8 and subsequent LPS stimulation. (B) Relative mRNA levels of *TNF* in cells (left) and protein level of TNF-α in culture medium (right) of U937 cells after addition of different concentrations of IL-8 and LPS stimulation. (C) Relative mRNA and protein levels of IL-6, (D) IL-8, and (E) TNF-α after treatment with different concentrations of VPA and LPS for 6 hours. For (A) and (B), data represent mean ± SD from at least two independent experiments (individual points shown) and are normalized to U937 cells without externally added IL-8. Statistical significance was assessed using one-way ANOVA followed by the Holm–Šidák multiple-comparisons test. Asterisks indicate significant differences relative to U937 cells without externally added IL-8. For (C–E), data represent mean ± SD from at least four independent experiments (individual points shown) and are normalized to U937 cells without VPA treatment. Statistical significance was assessed using one-way ANOVA followed by the Holm–Šidák multiple-comparisons test. Asterisks indicate significant differences relative to U937 cells without externally added VPA. *$p < 0.05$; **$p < 0.01$; ***$p < 0.001$. VPA, valproic acid

A

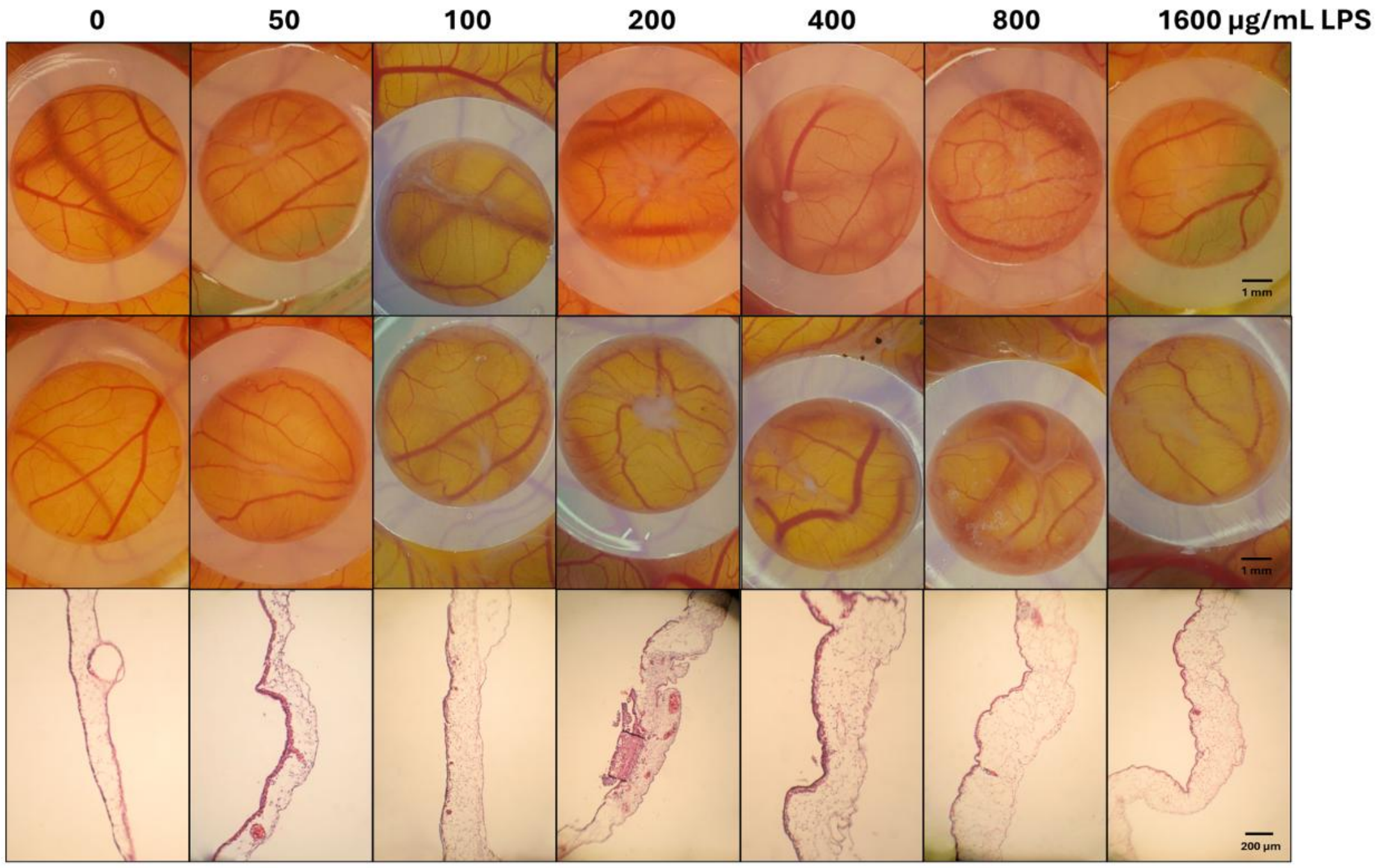

LPS concentration [µg/mL]	0	50	100	200	400	800	1600
Live/total number per group	22/23	22/23	20/21	24/24	21/23	20/24	13/26
Survival percentage [%]	95,7	95,7	95,2	100	91,3	83,3	50

B

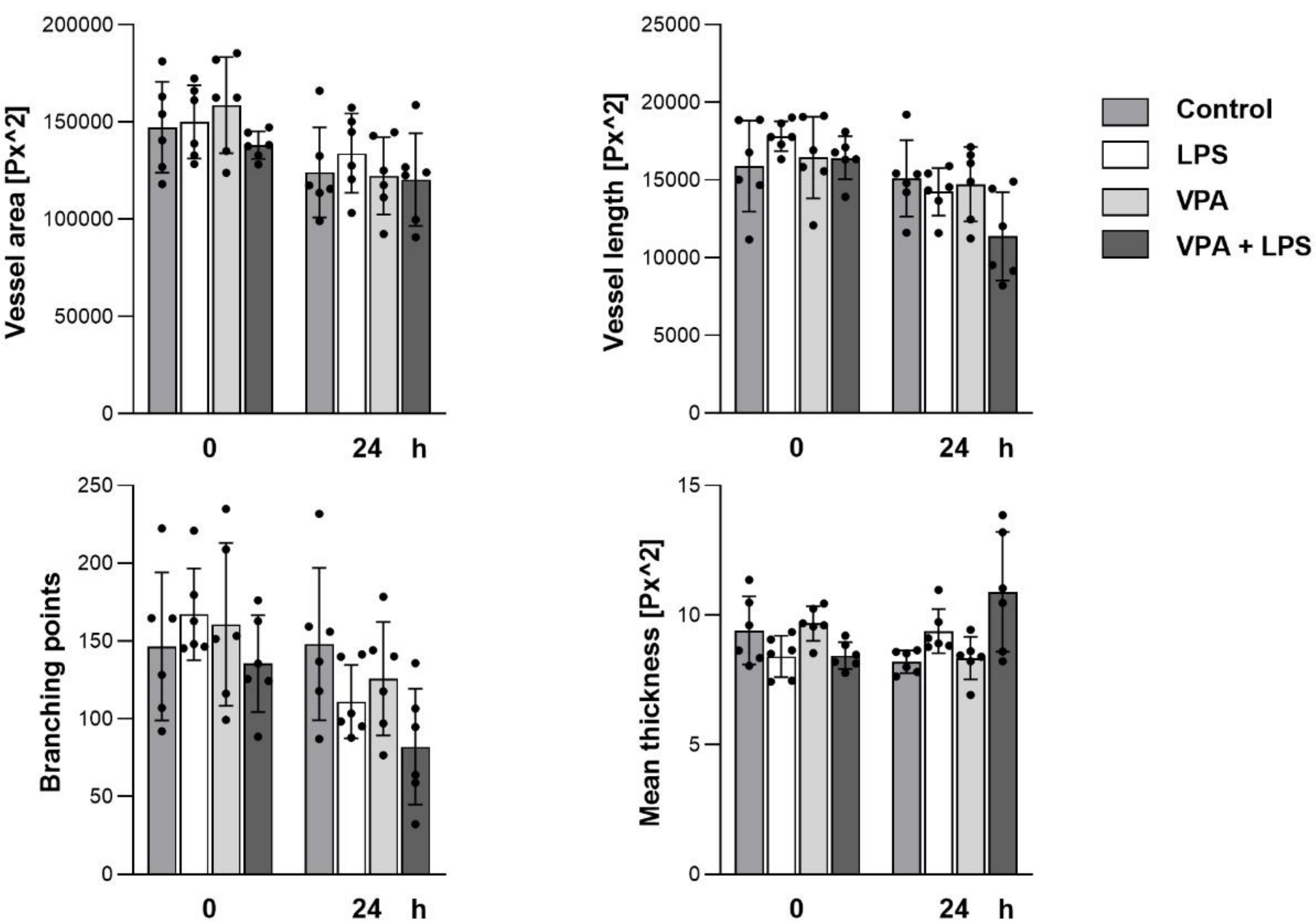

Figure S8. Effect of VPA treatment on viability and vascularization of the quail CAM

Japanese quail (*Coturnix japonica*) eggs were incubated at 37.5 °C and 50–60% humidity without rotation. On embryonic day 8 (ED8), silicone rings (6 mm diameter) were placed on the chorioallantoic membrane (CAM) to define treatment areas. On ED9, inflammation was induced by the application of different concentrations of LPS. Vasculature was analyzed using IKOSA Prisma® software (Kolaido) from digital photographs of CAM surface. Parameters of total vasculature area, total vasculature length, average vessel thickness and number of branching points were evaluated on the selected regions of interest. (A) Representative images of CAM show vascular architecture and the formation of white membrane deposits in response to increasing concentrations of LPS after 24 hours (two upper panels). Representative histological sections of CAM (bottom panels) illustrate tissue morphology and inflammatory changes, including preservation of epithelial layers and vasculature in untreated CAM compared with pronounced inflammatory cell infiltration in LPS-treated samples. Scale bars, 1 mm (CAM images) and 200 μm (histological sections). (B) Embryo survival following treatment with different concentrations of LPS is summarized in the table, showing the number of live embryos per total and the survival percentage. (C) Quantitative analysis of CAM vascularization parameters, including vessel area, vessel length, number of branching points, and mean vessel thickness, measured at 0 h and 24 h in samples treated with 100 μg/mL LPS and 2.5 mM VPA. Data represent mean ± SD from six CAM samples in each group (individual data points shown). Statistical significance was assessed using one-way ANOVA followed by the Holm–Šidák multiple-comparisons test.

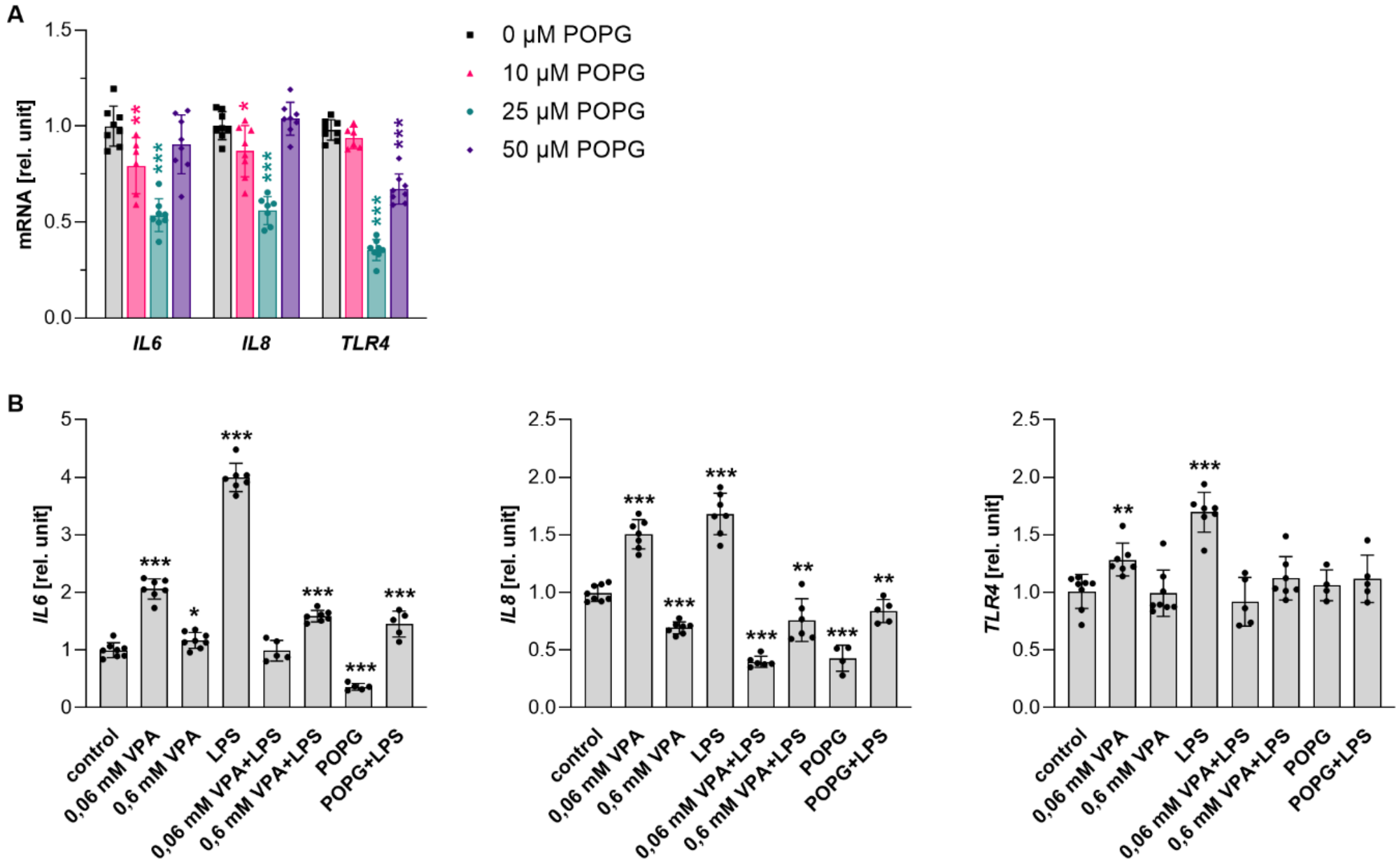

Figure S9. VPA treatment and POPG attenuate inflammation in quail CAM

Japanese quail (*Coturnix japonica*) eggs were incubated at 37.5 °C and 50–60% humidity without rotation. On embryonic day 8 (ED8), silicone rings (6 mm diameter) were placed on the chorioallantoic membrane (CAM) to define treatment areas. (A) On ED9, CAMs were stimulated with LPS (1 µg/mL) in the presence of increasing concentrations of POPG (0, 10, 25, or 50 µM) for 2 h. (B) VPA (0, 0.06, or 0.6 mM in saline) was applied for 24 h. On ED9, LPS (1 µg/mL), POPG (25 µM), or their combination was applied to the same area for 2 hours for RT–PCR analysis. Relative mRNA levels of *IL6*, *IL8*, and *TLR4* were determined in CAM tissue within the area defined by the silicone ring. Data in (A) are normalized to LPS-treated CAMs (0 µM POPG), whereas data in (B) are normalized to untreated CAM (0 mM VPA, 0 µg/mL LPS) and represent mean ± SD from at least five independent experiments (individual data points shown). Statistical significance was assessed using one-way ANOVA followed by the Holm–Šidák multiple-comparisons test. Asterisks indicate significant differences relative to the corresponding control group. *$p < 0.05$; **$p < 0.01$; ***$p < 0.001$. LPS, lipopolysaccharide; POPG, palmitoyl-oleoyl phosphatidylglycerol; VPA, valproic acid

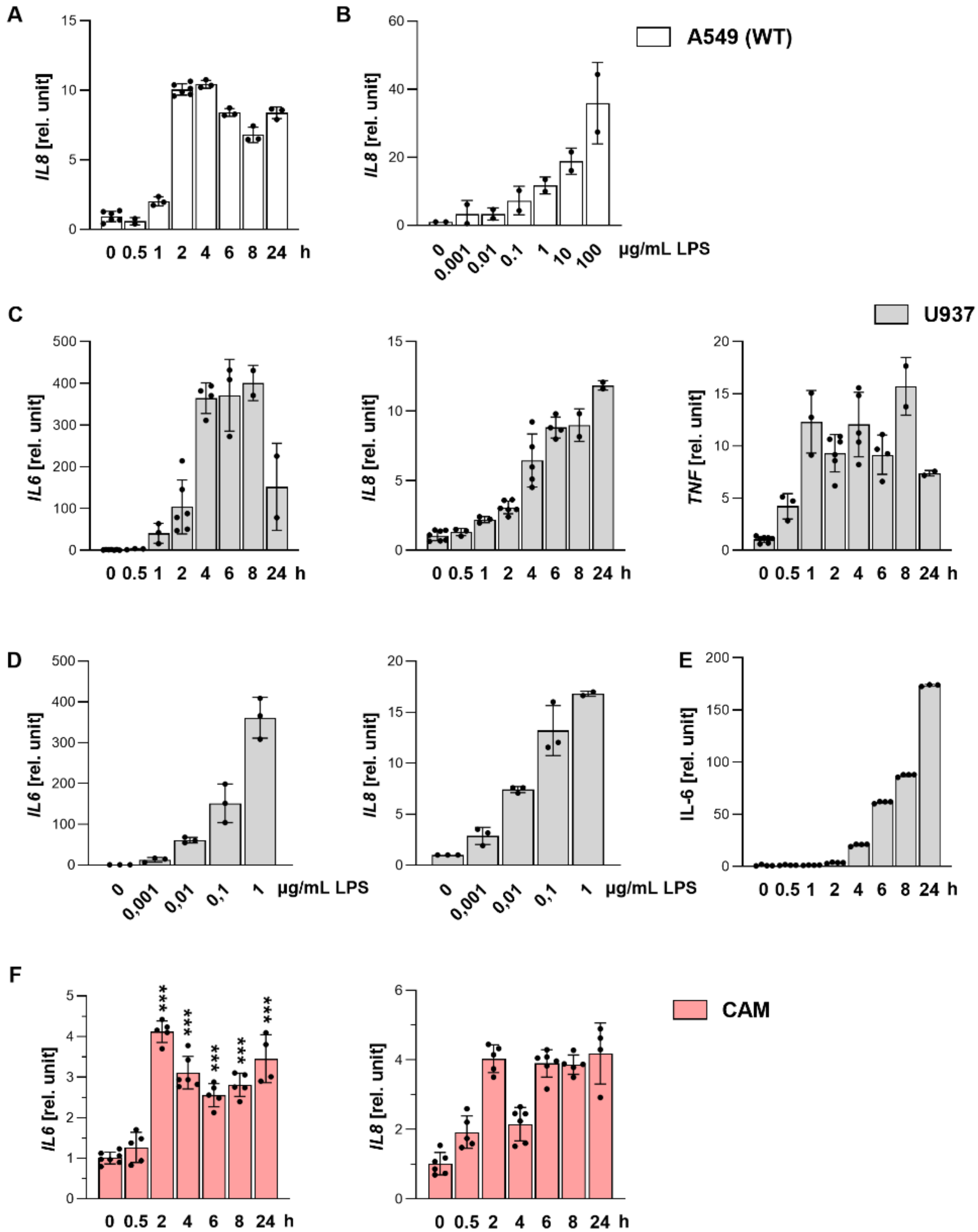

Figure S10. Optimization of experimental conditions for monitoring LPS-induced inflammation

(A, B) A549 cells were cultured in DMEM for 5 days and subsequently for an additional 2 days in DMEM lacking FBS. (A) Inflammation in A549 cells was induced by the addition of 1 µg/mL LPS to the culture medium, and the relative mRNA level of *IL8* was measured at the indicated time points. (B) Relative mRNA

levels of *IL8* were determined in cells stimulated with different concentrations of LPS for 2 hours. Data in (A) and (B) represent mean ± SD from at least two independent experiments (individual data points shown) and are normalized to untreated A549 cells (0 μg/mL LPS). Statistical significance was assessed using one-way ANOVA followed by the Holm–Šidák multiple-comparisons test. Asterisks indicate significant differences relative to untreated A549 cells. (C–E) Differentiation of U937 cells into macrophages was induced by seeding cells at 1×10^6 cells/mL and treating them with PMA (10 ng/mL) for 48 hours. Non-adherent cells were removed by washing with PBS, and adherent cells were cultured in fresh medium for an additional 24 hours. (C) Inflammation was induced by adding 1 μg/mL LPS, and the relative mRNA levels of *IL6*, *IL8*, and *TNF* were measured at the indicated time points. (D) Relative mRNA levels of *IL6* and *IL8* were determined after stimulation with different concentrations of LPS for 6 hours. (E) The relative protein level of IL-6 was measured in culture media of U937 cells stimulated with 0.1 μg/mL LPS at the indicated time points. Data in (C–E) represent mean ± SD from at least two independent experiments (individual data points shown) and are normalized to untreated U937 cells (0 μg/mL LPS). Statistical significance was assessed using one-way ANOVA followed by the Holm–Šidák multiple-comparisons test. Asterisks indicate significant differences relative to untreated U937 cells. (F) Japanese quail eggs were incubated at 37.5 °C and 50–60% humidity without rotation. On embryonic day 8 (ED8), silicone rings (6 mm diameter) were placed on the chorioallantoic membrane (CAM) to define treatment areas. Inflammation in CAM tissue was induced by adding 100 μg/mL LPS within the area defined by the silicone ring, and relative mRNA levels of *IL6* and *IL8* were determined at the indicated time points. Data are normalized to untreated CAM (0 μg/mL LPS) and represent mean ± SD from at least five independent experiments (individual data points shown). Statistical significance was assessed using one-way ANOVA followed by the Holm–Šidák multiple-comparisons test. Asterisks indicate significant differences relative to untreated CAM. *$p < 0.05$; **$p < 0.01$; ***$p < 0.001$. LPS, lipopolysaccharide

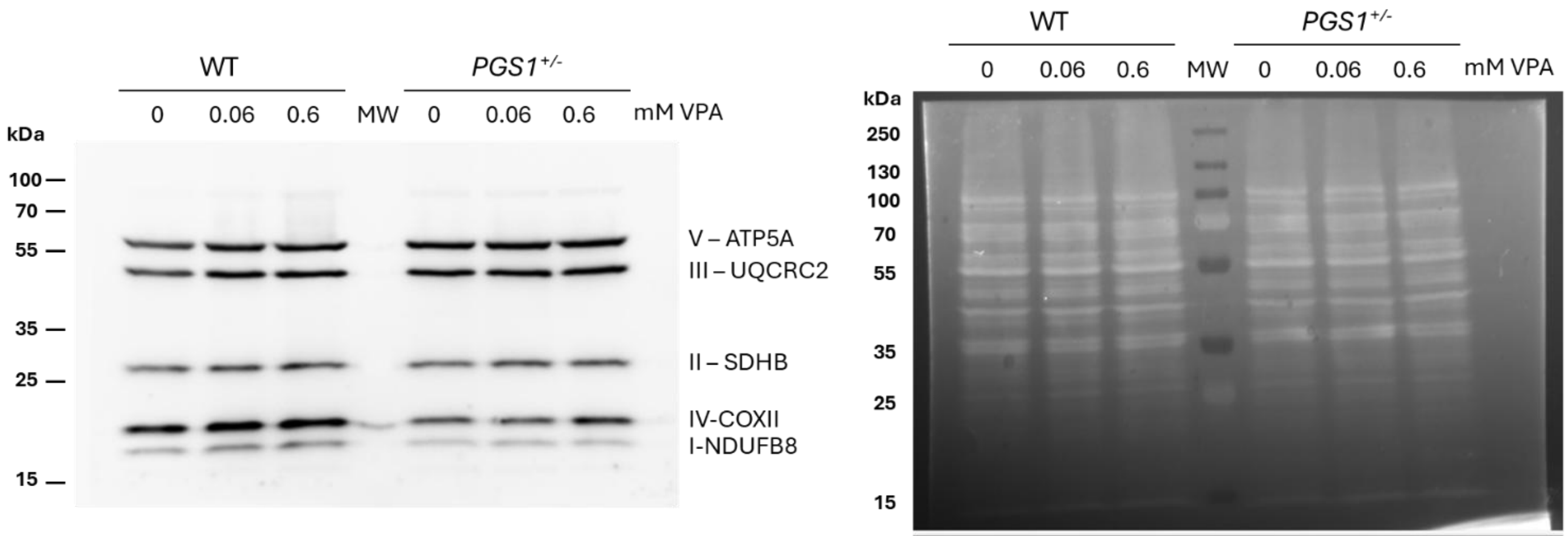

Data S1: Original Western blot images showing respiratory chain and ATP synthase subunits.

The original chemiluminescence images used for the quantification shown in Figure 1H are presented on the left. The corresponding No-Stain total protein images acquired from the same membranes prior to immunodetection are shown on the right and were used for normalization of protein abundance. Molecular weight markers are visible in the No-Stain images. Proteins detected with the OxPhos Human WB Antibody Cocktail correspond to ATP5A (Complex V), UQCRC2 (Complex III), SDHB (Complex II), MT-CO2/COXII (Complex IV), and NDUFB8 (Complex I).
